# The Impact of Hospital Volume on Bariatric Surgery Safety: A Population-Based Study using Data from the National Bariatric Surgery Registry of the United Kingdom

**DOI:** 10.1007/s11695-026-08946-1

**Published:** 2026-09-22

**Authors:** Stergios Bobotis, Bibek Das, Henry Robb, Chetan Parmar, Yasser Abdul Aal, Nour El Ghazal, Hutan Ashrafian, Omar M. Ghanem, Emma Rose McGlonne, Omar Khan, Matyas Fehervari

**Affiliations:** 1 Department of Surgery, Maidstone and Tunbridge Wells NHS Trust, Maidstone, UK; 2 Department of Surgery and Cancer, Imperial College London, London, UK; 3 Department of General Surgery, Chelsea and Westminster Hospital NHS Foundation Trust, London, UK; 4 Department of Surgery, Whittington Health NHS Trust, London, UK; 5 St George’s, University of London, London, UK; 6 Canterbury Christ Church University, Canterbury, UK

## Abstract

**Background:**

Hospital procedural volume is recognised as an important determinant of outcomes in complex surgery. However, the volume–outcome relationship in bariatric surgery remains incompletely defined. This study examined the association between hospital procedural volume and major complications in primary metabolic bariatric surgery using the United Kingdom (UK) National Bariatric Surgery Registry (NBSR).

**Methods:**

Prospectively collected NBSR data from 2014 to 2023 were analysed. Adults undergoing RYGB, SG, or OAGB were included. Hospitals were stratified into quartiles using a four-year moving average of annual procedural volume. Primary outcomes were major postoperative complications, including gastrojejunal obstruction, bleeding, and Clavien-Dindo grade ≥ III events. Multivariable logistic regression assessed associations between hospital volume and outcomes.

**Results:**

A total of 54,195 patients were included (RYGB: 23,139; SG: 25,300; OAGB: 4,744). Complication rates were low overall. For RYGB, obstruction rates declined with increasing hospital volume (0.8% in quartile I vs. 0.2% in quartile IV; *p* < 0.001). Higher volume independently predicted lower risk of obstruction (OR 0.31, 95% CI 0.16–0.58; *p* < 0.001) and Clavien-Dindo ≥ III complications (OR 0.71, 95% CI 0.51–0.99; *p* = 0.044). No significant associations between volume and outcomes were observed for SG or OAGB.

**Conclusion:**

Higher hospital volume was associated with fewer severe complications following RYGB, although complication rates remained low across all volume quartiles. Outcomes for SG and OAGB were consistently favourable across all volume quartiles, indicating a lesser influence of hospital volume. These findings support continued evaluation of minimum volume standards within bariatric accreditation frameworks.

**Supplementary Information:**

The online version contains supplementary material available at https://doi.org/10.1007/s11695-026-08946-1.

## Introduction

 Metabolic bariatric surgery (MBS) is an established intervention for adults with severe obesity, offering substantial and sustained improvements in weight, obesity-related comorbidities, and health-related quality of life [1, 2]. It has a safety profile comparable to other common surgical procedures, as demonstrated by analyses of the American College of Surgeons National Surgical Quality Improvement Program (ACS-NSQIP) [3]. Laparoscopic bariatric surgery is considered technically complex, where advanced training is associated with lower complication rates [4, 5].

In the United Kingdom, the National Bariatric Surgery Registry (NBSR) serves as the official national registry, governed by the NBSR committee and supported by the British Obesity and Metabolic Surgery Society (BOMSS). It was designed to monitor patient outcomes, institutional performance, and clinical safety across UK bariatric surgery units. By capturing detailed perioperative data, the NBSR supports audit, benchmarking, and service improvement across high- and low-volume centres [6].

Prior studies, although variable in the registries used — including University HealthSystem Consortium (UHC) and National Inpatient Sample (NIS) — have suggested that high-volume or accredited centres achieve lower morbidity and mortality [7]. It was shown that a 10-case increment increase in hospital and surgeon volume resulted in a lower odd of 90-day morbidity after RYGB (OR = 0.86 for hospital volume and OR = 0.82 for surgeon volume, *p* < 0.05). Reported thresholds vary, with some analyses identifying potential benefits above 20–25 annual RYGB cases per surgeon or centre, while others suggest diminishing returns beyond approximately 250 annual cases [8–10]. These studies, however, are largely US-based, with some limited to state-level datasets, and also rely on administrative or general surgical registries which may not be directly generalizable to other healthcare systems, where the degree of centralization, case selection criteria, and perioperative care pathways can vary substantially.

Similar volume–outcome patterns have been observed in upper gastrointestinal surgery, where centralization of pancreatic and esophageal procedures has been associated with improved survival in high-volume centres [11–13]. Whether these findings apply to metabolic bariatric surgery remains debated [7]. Evidence supporting improved long-term outcomes through centralization is limited, and some argue that appropriately equipped regional hospitals may achieve comparable results [14]. Moreover, centralization may introduce access barriers for patients in rural or socioeconomically disadvantaged regions. Travel distance, financial burden, and social isolation may limit access to care and exacerbate disparities [15, 16].

In recognition of these associations, early quality initiatives promoted the concept of “Centers of Excellence,” encouraging referral to institutions with specialized infrastructure and greater procedural experience [17, 18]. Although some studies suggested that accreditation led to better outcomes [19, 20], others questioned whether structural designation alone—without accounting for volume or outcomes—was sufficient [21, 22]. This led to the development of the Metabolic and Bariatric Surgery Accreditation and Quality Improvement Program (MBSAQIP), a joint initiative by the American College of Surgeons and the American Society for Metabolic and Bariatric Surgery. MBSAQIP integrates risk-adjusted outcomes, multidisciplinary standards, and minimum procedural volume requirements—mandating at least 50 stapled cases annually for comprehensive centres, with additional recommendations for anastomotic volume thresholds [23].

This study aims to examine the association between hospital volume and postoperative complication rates following primary RYGB, SG, and one-anastomosis gastric bypass (OAGB), using national registry data from nearly a decade of UK metabolic bariatric practice.

## Methods

### Study Design and Data Source

This was a national database analysis of prospectively collected registry data from the United Kingdom National Bariatric Surgery Registry (UK NBSR) for patients undergoing bariatric surgery between January 1, 2014, and August 4, 2023. Patient-level data were extracted from three main datasets: baseline characteristics, procedural data, and complication records. All data were anonymized in accordance with applicable data governance regulations, and data collection was conducted in compliance with the principles of the Declaration of Helsinki.

Eligible patients included those who underwent a primary Roux-en-Y gastric bypass (RYGB), sleeve gastrectomy (SG), or one anastomosis gastric bypass (OAGB). Only first-time anatomical bariatric operations were considered primary. Procedures performed following a gastric balloon were also classified as primary surgeries. In contrast, operations performed after a prior gastric band, gastric bypass, sleeve, or any other anatomical bariatric procedure were considered revisional and were excluded from all primary surgery analyses. Non-anatomical procedures, including intragastric balloon placement, endoscopic sleeve gastroplasty, and EndoBarrier, were excluded from the study. Surgery type was determined using the recorded operation field, while primary or revisional status was dynamically derived within each surgical cohort based on procedure chronology and prior operation history. Hospitals with fewer than 10 procedures were excluded from the analysis.

Each patient was assigned a unique PatientID, and each operation was identified by a unique EntryID. Additional information collected included age at operation, gender, ethnic origin, hospital, funding category, body mass index (BMI), American Society of Anesthesiology (ASA) grade, type 2 diabetes mellitus (T2DM), co-morbidities, functional status, operation type, date of discharge, date of complication, type of complication, and management.

### Hospital Volume Calculation

To evaluate the effect of hospital volume on outcomes, patients were stratified into four quartiles of equal size based on hospital volume. Hospital volume was calculated using a 4-year moving average to account for temporary fluctuations in annual hospital activity and incorporate the cumulative experience achieved over time. For each operation, the moving average volume included the year of operation and the preceding three years. For example, the hospital volume for a patient undergoing surgery in 2005 was calculated as the mean annual volume of procedures at that hospital for the years 2002, 2003, 2004, and 2005. For earlier years with incomplete data, the available years were included in the calculation. This method was adapted from Markar et al. [24].

### Outcome Measures

The primary outcomes were major postoperative complications, including obstruction, bleeding, leak, Clavien-Dindo grade ≥ III (CDIII+), and venous thromboembolism (VTE). Obstruction was defined as any post-gastrojejunal obstruction and was further categorized by cause, including adhesions, and internal hernia. Leak was defined as evidence of postoperative spillage of gastrointestinal content, including but not limited to collections or free air around the anastomosis or stapler line, based on imaging or intraoperative findings.

Covariates included in the multivariable analyses were age at operation, gender, BMI immediately prior to surgery, T2DM, hypertension, cardiovascular disease (CVD), obstructive sleep apnea (OSA), and functional status. OSA and functional status were collapsed into binary categories where necessary due to low event frequencies.

### Statistical Analysis

Continuous variables were summarized as medians with interquartile ranges (IQR), and categorical variables were reported as frequencies and percentages. Comparisons of categorical data were performed using Pearson’s chi-square test. The Kruskal-Wallis rank-sum test was used to compare non-parametric continuous data across multiple groups. Pairwise comparisons between hospital volume quartiles were performed for each complication where possible using Bonferroni-adjusted proportion tests. The absolute risk of complications was calculated for each moving average hospital volume by dividing the number of obstruction cases by the total number of patients. Multivariable logistic regression models assessed the association between hospital volume quartiles and postoperative complications, adjusting for demographic and clinical covariates (age, sex, BMI, type 2 diabetes mellitus, hypertension, cardiovascular disease, sleep apnea, functional status, and funding category). Odds ratios (OR) and 95% confidence intervals (CI) were reported. All statistical analyses were performed using R 4.5.0 (R Foundation for Statistical Computing, Vienna, Austria).

### Funding

No funding was used for this article.

## Results

### Baseline Characteristics

A total of 54,280 patients underwent primary metabolic bariatric surgery, comprising 24,139 Roux-en-Y gastric bypass (RYGB), 25,397 sleeve gastrectomy (SG), and 4,744 one-anastomosis gastric bypass (OAGB) procedures. Median annual hospital volume increased substantially across quartiles for all primary surgeries, from 39 cases (IQR 26–53) in quartile I to 263 cases (IQR 232–308) in quartile IV. This pattern was consistent across procedures: RYGB ranged from 42 (IQR 27–59) to 280 (IQR 249–320), SG from 36 (IQR 23–57) to 283 (IQR 250–305), and OAGB from 54 (IQR 30–62) to 287 (IQR 260–342). Demographic characteristics were consistent across quartiles. Median age ranged between 45 and 46 years, while the proportion of female patients declined with increasing volume (82% in quartile I vs. 79% in quartile IV. Markers of clinical complexity differed by hospital volume. The prevalence of insulin-treated diabetes increased from 4.1% to 6.6% across quartiles, while the proportion of patients classified as ASA grade III or IV rose from 33% to 42%. These trends were observed across all procedure types, with the most pronounced differences noted in the RYGB cohort. Although the proportion of patients classified as ASA grade III or IV increased with hospital volume, median preoperative BMI was 44 kg/m² in every quartile. ASA grade and BMI reflect different dimensions of case mix, and the ASA distribution should not be interpreted as a surrogate for the prevalence of BMI > 40 kg/m² (Table 1).

**Table 1:** Baseline characteristics for of all primary bariatric surgeries (RYGB, SG, OAGB) by hospital volume quartile

Characteristic	Overall *N* = 54,195^1^	I *N* = 13,549^1^	II *N* = 13,549^1^	III *N* = 13,549^1^	IV *N* = 13,548^1^
Annual volume	120 (66, 194)	39 (26, 53)	90 (77, 104)	150 (131, 174)	263 (232, 308)
Hospital count	198	99	55	29	15
Age	45 (36, 53)	45 (36, 53)	45 (36, 53)	46 (36, 54)	46 (36, 54)
Gender					
Female	43,325 (80%)	10,916 (81%)	10,999 (81%)	10,726 (79%)	10,684 (79%)
Male	10,870 (20%)	2,633 (19%)	2,550 (19%)	2,823 (21%)	2,864 (21%)
Pre-op BMI	44 (40, 49)	44 (40, 49)	44 (40, 49)	44 (40, 50)	44 (40, 49)
Diabetic					
Injectable other than insulin	628 (1.2%)	187 (1.4%)	167 (1.2%)	157 (1.2%)	117 (0.9%)
Insulin treatment	2,888 (5.3%)	556 (4.1%)	636 (4.7%)	803 (5.9%)	893 (6.6%)
No indication of type 2 diabetes	40,030 (74%)	10,483 (77%)	10,245 (76%)	9,706 (72%)	9,596 (71%)
Oral hypoglycaemics	8,331 (15%)	1,796 (13%)	1,973 (15%)	2,195 (16%)	2,367 (17%)
Pre-diabetes	2,318 (4.3%)	527 (3.9%)	528 (3.9%)	688 (5.1%)	575 (4.2%)
ASA grade					
ASA I	3,463 (6.6%)	935 (7.1%)	902 (6.9%)	957 (7.2%)	669 (5.0%)
ASA II	28,930 (55%)	7,768 (59%)	6,755 (52%)	7,265 (55%)	7,142 (53%)
ASA III	20,106 (38%)	4,364 (33%)	5,271 (40%)	4,994 (38%)	5,477 (41%)
ASA IV	319 (0.6%)	78 (0.6%)	90 (0.7%)	62 (0.5%)	89 (0.7%)
Length of stay	2.00 (1.00, 2.00)	2.00 (1.00, 2.00)	2.00 (1.00, 2.00)	2.00 (1.00, 2.00)	2.00 (1.00, 2.00)

### Complications

**Fig. 1:**
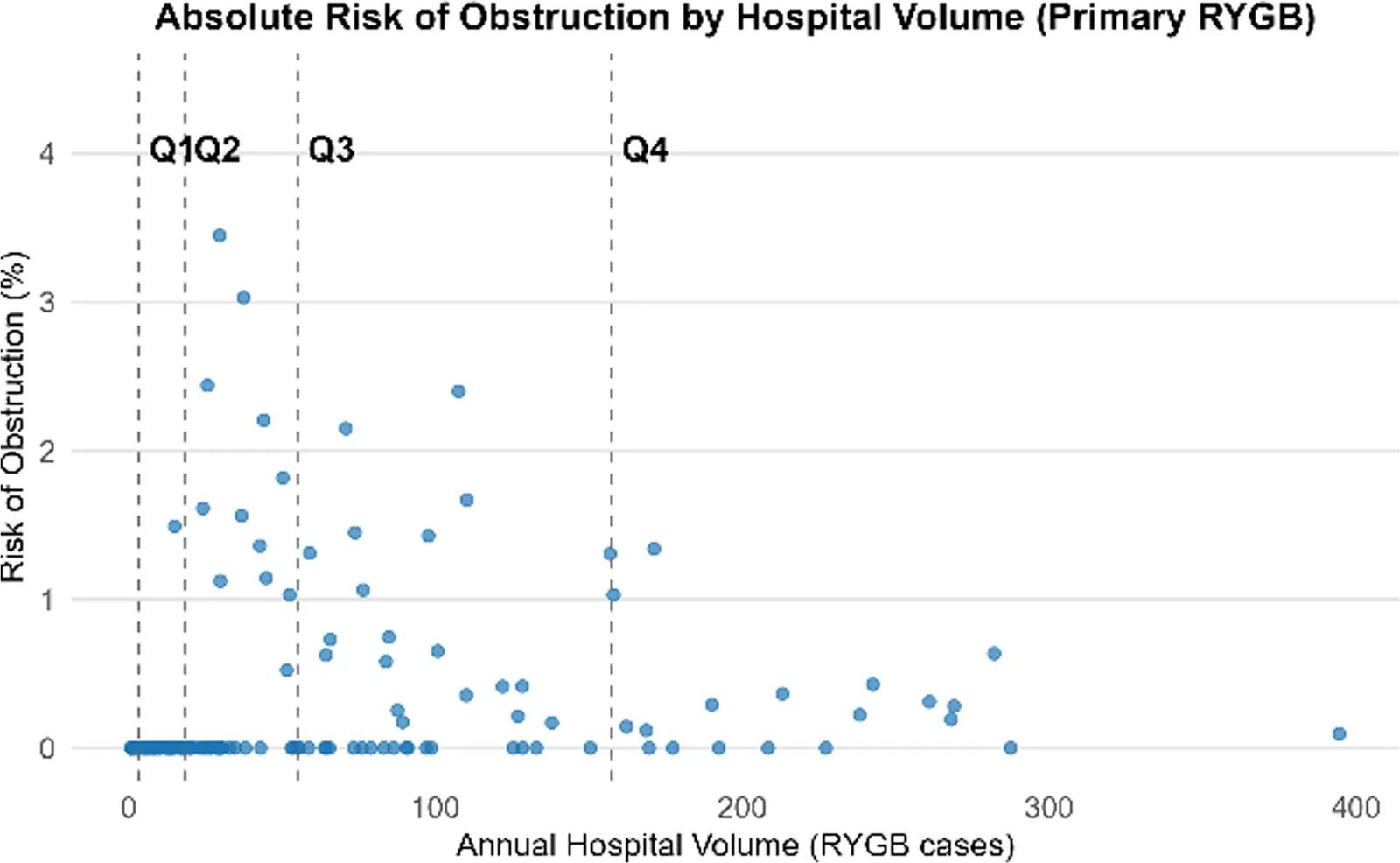
Absolute risk of postoperative obstruction by annual hospital volume after primary RYGB. Each point represents the observed obstruction rate at a given four-year moving-average hospital volume; dashed vertical lines indicate quartile boundaries

**Fig. 2:**
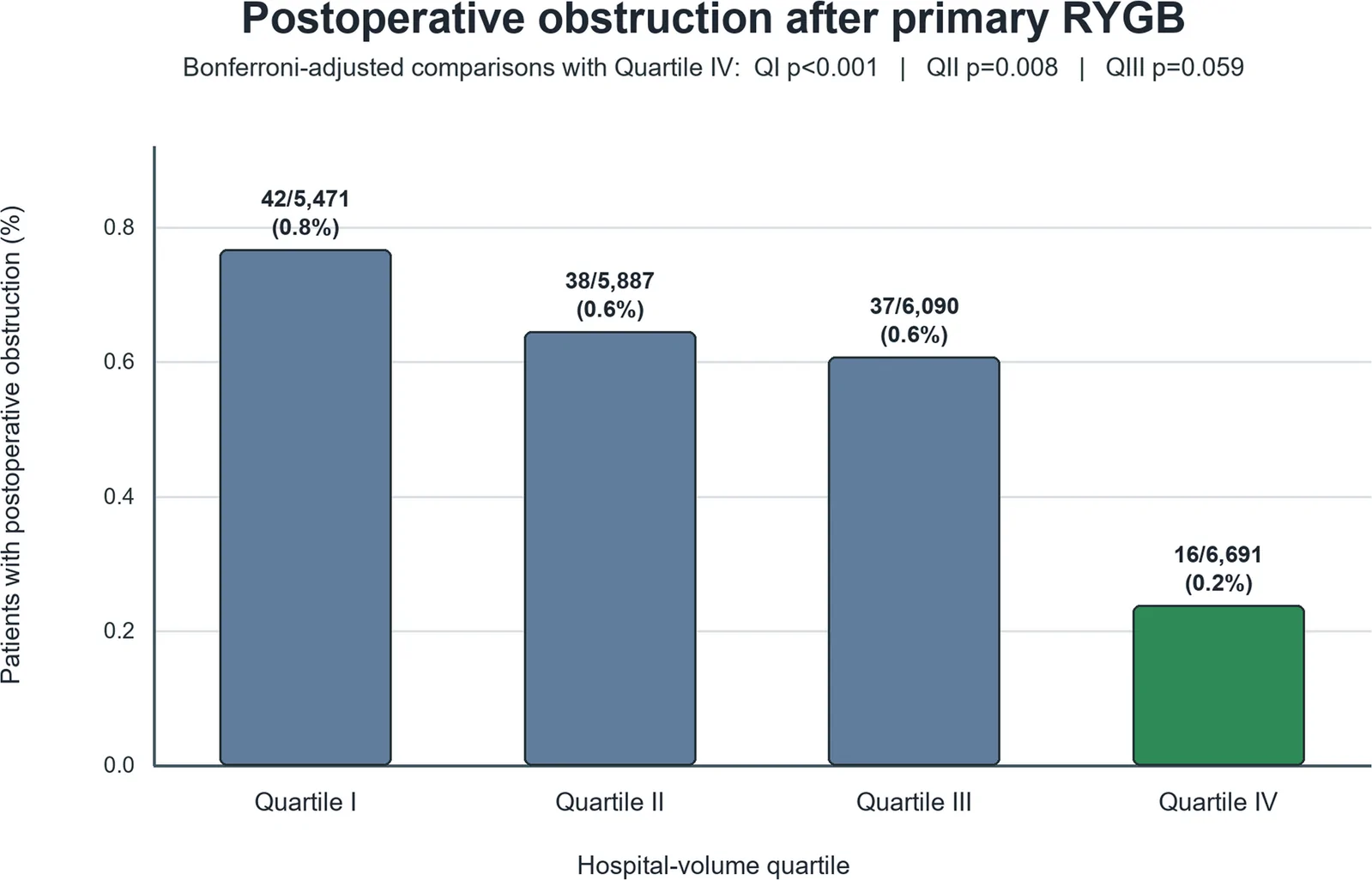
Postoperative obstruction following primary RYGB by hospital-volume quartile. Bars show observed proportions; labels show events/total patients and percentages. Bonferroni-adjusted pairwise comparisons with Quartile IV were *p* < 0.001 for Quartile I, *p* = 0.008 for Quartile II, and *p* = 0.059 for Quartile III

The overall rate of any complication regardless of CD severity after RYGB was low at 2.2% (531/24,139), with no significant difference across hospital volume quartiles (*p* = 0.089). Leak complications occurred in 0.5% of cases, with no significant difference across volume groups (*p* = 0.625). Obstruction rates were significantly lower in higher-volume centres, decreasing from 0.8% in quartile I to 0.2% in quartile IV (p = < 0.001). Figure 1 shows the absolute risk of obstruction across the range of annual hospital volume. Figure 2 presents obstruction rates by hospital-volume quartile and the relevant Bonferroni-adjusted pairwise comparisons; rates were lower in Quartile IV than in Quartiles I (*p* < 0.001) and II (*p* = 0.008), while the comparison with Quartile III did not meet the adjusted significance threshold (*p* = 0.059). Obstruction causes were heterogeneous, most commonly attributed to anastomotic anatomy (35/133, 26%) and other unspecified etiologies (36/133, 27%), with smaller numbers due to adhesions (11/133, 8%) and internal hernia (16/133, 12%) (Table S2). Bleeding (0.7%), and Clavien-Dindo grade ≥ III complications (1.4%) were comparable across quartiles (*p* > 0.15). Stricture and VTE occurred in < 0.1% of cases (Table 2).

**Table 2:** Complication rates by hospital volume quartile for (a) all primary procedures, (b) primary RYGB, and (c) primary SG

Characteristic	Overall *N* = 54,280^1^		I *N* = 13,569^1^	II *N* = 13,581^1^			III *N* = 13,570^1^		IV *N* = 13,560^1^	*p*-value^2^
Leak	151 (0.3%)		33 (0.2%)	36 (0.3%)			37 (0.3%)		45 (0.3%)	0.551
Bleeding	196 (0.4%)		43 (0.3%)	53 (0.4%)			50 (0.4%)		50 (0.4%)	0.777
Obstruction	142 (0.3%)		44 (0.3%)	39 (0.3%)			41 (0.3%)		18 (0.1%)	0.008
Stricture	20 (< 0.1%)		10 (< 0.1%)	5 (< 0.1%)			2 (< 0.1%)		3 (< 0.1%)	0.072
Nutrition Failure	2 (< 0.1%)		0 (0%)	0 (0%)			2 (< 0.1%)		0 (0%)	0.187
Reoperation	549 (1.0%)		154 (1.1%)	134 (1.0%)			136 (1.0%)		125 (0.9%)	0.355
CDIII+	375 (0.7%)		93 (0.7%)	99 (0.7%)			101 (0.7%)		82 (0.6%)	0.508
VTE	25 (< 0.1%)		2 (< 0.1%)	8 (< 0.1%)			15 (0.1%)		0 (0%)	< 0.001
Roux-en-Y Gastric Bypass										
Characteristic	Overall *N* = 24,139^1^	I *N* = 5,471^1^			II *N* = 5,887^1^	III *N* = 6,090^1^		IV *N* = 6,691^1^		p-value^2^
Leak	126 (0.5%)	25 (0.5%)			28 (0.5%)	32 (0.5%)		41 (0.6%)		0.625
Bleeding	178 (0.7%)	41 (0.7%)			46 (0.8%)	46 (0.8%)		45 (0.7%)		0.902
Obstruction	133 (0.6%)	42 (0.8%)			38 (0.6%)	37 (0.6%)		16 (0.2%)		< 0.001
Stricture	18 (< 0.1%)	9 (0.2%)			4 (< 0.1%)	2 (< 0.1%)		3 (< 0.1%)		0.070
Nutritional Failure	2 (< 0.1%)	0 (0%)			0 (0%)	2 (< 0.1%)		0 (0%)		0.174
Reoperation	378 (1.6%)	98 (1.8%)			97 (1.6%)	97 (1.6%)		86 (1.3%)		0.138
CDIII+	345 (1.4%)	86 (1.6%)			92 (1.6%)	90 (1.5%)		77 (1.2%)		0.150
VTE	4 (< 0.1%)	0 (0%)			2 (< 0.1%)	2 (< 0.1%)		0 (0%)		0.266
All Complications	531 (2.2%)	134 (2.4%)			140 (2.4%)	134 (2.2%)		123 (1.8%)		0.089
Sleeve Gastrectomy										
Characteristic	Overall *N* = 25,397^1^	I *N* = 7,281^1^			II *N* = 6,519^1^	III *N* = 6,163^1^		IV *N* = 5,434^1^		p-value^2^
Leak	16 (< 0.1%)	6 (< 0.1%)			4 (< 0.1%)	4 (< 0.1%)		2 (< 0.1%)		0.835
Bleeding	4 (< 0.1%)	1 (< 0.1%)			0 (0%)	1 (< 0.1%)		2 (< 0.1%)		0.422
Obstruction	3 (< 0.1%)	1 (< 0.1%)			0 (0%)	2 (< 0.1%)		0 (0%)		0.397
Stricture	2 (< 0.1%)	1 (< 0.1%)			1 (< 0.1%)	0 (0%)		0 (0%)		> 0.999
Reoperation	158 (0.6%)	55 (0.8%)			32 (0.5%)	36 (0.6%)		35 (0.6%)		0.251
CDIII+	19 (< 0.1%)	6 (< 0.1%)			3 (< 0.1%)	8 (0.1%)		2 (< 0.1%)		0.267
VTE	21 (< 0.1%)	2 (< 0.1%)			6 (< 0.1%)	13 (0.2%)		0 (0%)		< 0.001
All Complications	188 (0.7%)	60 (0.8%)			40 (0.6%)	51 (0.8%)		37 (0.7%)		0.389

^1^n – patient count (%)

^2^Pearson’s Chi-squared test; Fisher’s exact test

For SG, complication rates were similarly low and the composite outcome of all complications did not differ significantly by volume (0.7% overall; *p* = 0.389). Leak, bleeding, obstruction, stricture, Clavien-Dindo grade ≥ III complications or VTE were observed in < 0.1% of the population.

Complications following OAGB were rare and comparable across quartiles. Bleeding occurred in 0.3% of patients, and obstruction in 0.1%. No cases of VTE or stricture were recorded. Leak events occurred in 0.2%. CDIII+ complications were observed in 0.3% of cases with no significant differences across quartiles (*p* = 0.852) (Table S1).

### Logistic Regression Analysis

In multivariable logistic regression analysis, hospital volume was independently associated with reduced odds of postoperative obstruction following RYGB, with significantly lower odds observed in quartile IV compared to quartile I (OR 0.31, 95% CI 0.16–0.58; p = < 0.001). No significant associations were found between hospital volume and leak complications in either RYGB or SG. For CDIII+ complications, a statistically significant reduced risk was observed in RYGB patients treated in quartile IV centres when compared to Quartile I (OR 0.71, 95% CI 0.51–0.99; *p* = 0.044) (Table 3). No volume–outcome associations were observed for SG CDIII+ complications. Logistic regression was not performed for bleeding risk in SG and all complications in OAGB due to the small number of recorded complications (Tables S3-S7). The obstruction model is reported in Supplementary Table S3 and included BMI and major recorded comorbidities; BMI was not independently associated with obstruction (OR 0.99, 95% CI 0.97–1.00; *p* = 0.5).

**Table 3:** Postoperative complications by hospital volume quartile: multivariable logistic regression analysis

Surgery type	Outcome	Quartile	OR (95% CI)	*p*-value
RYGB	**Obstruction**	I	1.00 (reference)	
		II	0.93 (0.58–1.49)	0.874
		III	0.90 (0.56–1.46)	0.752
		IV	0.31 (0.16–0.58)	< 0.001
	**Leak**	I	1.00 (reference)	
		II	0.85 (0.42–1.72)	> 0.9
		III	0.98 (0.51–1.95)	> 0.9
		IV	1.02 (0.54–1.99)	0.541
	**CDIII+ Complications**	I	1.00 (reference)	
		II	0.98 (0.70–1.35)	0.821
		III	0.94 (0.68–1.30)	0.791
		IV	0.71 (0.51–0.99)	0.044
SG	**Leak**	I	1.00 (reference)	
		II	0.68 (0.17–2.39)	0.573
		III	0.65 (0.16–2.33)	0.581
		IV	0.37 (0.05–1.63)	0.234
	**CDIII+ Complications**	I	1.00 (reference)	
		II	0.64 (0.13–2.62)	0.511
		III	1.55 (0.51–5.26)	0.423
		IV	0.46 (0.07–2.19)	0.413

All multivariable logistic regression models were adjusted for age at operation, gender, BMI, type 2 diabetes mellitus, hypertension, cardiovascular disease, sleep apnoea, functional status, and funding

## Discussion

In this study, treatment at higher-volume hospitals was associated with a significantly reduced risk of postoperative obstruction following Roux-en-Y gastric bypass (RYGB). We observed a stepwise decline in absolute obstruction risk across increasing hospital volume quartiles. However, despite the statistically significant association, the absolute event rates remained low across all volume groups, decreasing from 0.8% in quartile I to 0.2% in quartile IV. Therefore, although the relative reduction in risk was substantial, the clinical relevance at the individual patient level should be interpreted cautiously. Nevertheless, postoperative obstruction remains an important complication that frequently requires reoperation and may result in significant morbidity, meaning that even small absolute reductions may be meaningful at a population level. Multivariable logistic regression confirmed hospital volume as an independent predictor of reduced obstruction and Clavien-Dindo grade ≥ III complications, the latter emerging only after adjustment for clinical covariates. In contrast, complication rates following sleeve gastrectomy and one-anastomosis gastric bypass were low overall and did not vary significantly by volume. However, given the lower event rates in these procedures, these analyses may have been underpowered to detect subtle differences. Median overall length of stay was 2 days in every hospital-volume quartile; however, this measure was not conditioned on the occurrence of obstruction and therefore cannot determine whether higher-volume centres achieve better rescue outcomes after an obstruction has occurred.

### High vs. Low-Volume Hospitals

Although significant differences between hospital volume groups were limited in our study, our findings are broadly consistent with previous literature suggesting that procedural volume may influence selected outcomes following complex gastrointestinal surgery [22, 25–29]. Early evidence established that high-volume hospitals consistently achieve lower complication and mortality rates, a pattern attributed to greater procedural expertise, institutional experience, and the adoption of standardized care pathways [28, 29]. The relevance of these associations has been confirmed on an international scale [24, 30]; for example, the NordOSCo study demonstrated significantly improved long-term survival after bariatric surgery in high-volume Nordic centres [24]. High-volume centres may also accelerate surgical learning curves and streamline credentialing, owing to concentrated procedural exposure, structured mentorship, and formalized training pathways. These mechanisms contribute to consistently improved outcomes and help maintain institutional proficiency over time [31]. Our results contribute to this evidence base by identifying postoperative obstruction as associated with hospital volume in RYGB procedures: obstruction occurred in 0.8% of Quartile I (42/5,471), 0.6% of Quartile II (38/5,887), 0.6% of Quartile III (37/6,090), and 0.2% of Quartile IV (16/6,691). Compared with Quartile IV, Bonferroni-adjusted p values were < 0.001 for Quartile I, 0.008 for Quartile II, and 0.059 for Quartile III; in the adjusted model, Quartile IV was associated with lower odds than Quartile I (OR 0.31, 95% CI 0.16–0.58; *p* < 0.001). While our findings suggest that hospital volume may influence selected outcomes following RYGB, the generally low complication rates observed across all volume quartiles indicate that high-quality bariatric care can be delivered across a broad range of UK centres.

### Accreditation

Our results also align with current accreditation standards, such as those set by the Metabolic and Bariatric Surgery Accreditation and Quality Improvement Program (MBSAQIP), which incorporate procedural volume as a key quality metric [32]; the minimum case volume needed to maintain accreditation was set as 50 stapling procedures a year; a threshold surpassed by hospitals in quartiles II–IV, which showed trends toward improved postoperative outcomes.

However, the independent effect of accreditation has been questioned. Studies by Dimick et al. and Birkmeyer et al. found no direct reduction in complications attributable to accreditation alone, suggesting that embedded factors such as hospital volume may be the true driver of improved outcomes [21, 29]. In contrast, Jafari et al. reported that accreditation was associated with better bariatric outcomes even after adjusting for volume [22]. Our findings suggest that hospital volume may contribute to variation in selected outcomes following RYGB. However, given the generally low complication rates and the limited differences observed across most outcomes, the relationship between accreditation, procedural volume, and bariatric outcomes is likely multifactorial and warrants further investigation.

### Surgical Skill and Expertise

Surgical skill itself, which strongly correlates with volume, has also been independently associated with outcomes [26], particularly in RYGB — a technically complex bariatric procedure owing to its anastomotic component. Surgeons with a 53 annual RYGB case load were associated with a lower summary skill quartile, which in turn was related to longer operative times and increased complication rates [5]. The opposite relationship has been established with a 157 annual surgeon volume for RYGB. Altieri et al. further support those findings by stating that a surgeon’s learning curve can be set to 50 annual cases, a volume deemed adequate for RYGB laparoscopic proficiency [9]. In our study, although surgeon-specific volume was not directly assessed, higher hospital volume was associated with significantly reduced rates of obstruction and Clavien-Dindo grade ≥ III complications. These findings may reflect differences in surgical expertise, operative technique, institutional experience, or perioperative care processes. However, the specific factors underlying the observed association cannot be determined from the present study.

### Multidisciplinary Teams (MDT)

Notably, centralization studies in oncology have reported a decline in surgical resection rates and broader use of palliative care, likely reflecting more nuanced multidisciplinary team (MDT) decision-making. While these shifts improve overall care, they also introduce potential selection bias through stage migration, where improved diagnostic precision can artificially enhance outcomes across staging categories [33].

In the bariatric setting, high-volume centres similarly benefit from robust MDT infrastructure, including psychological and medical risk assessment. These centres often adopt formalized audit processes and multidisciplinary perioperative care models that improve care coordination, patient selection, and adherence to best-practice pathways [34]. Additionally, they are more likely to implement Enhanced Recovery After Surgery (ERAS) protocols, which have been associated with reduced complication rates, improved pain control, and shorter hospital stays in bariatric populations [35]. However, our study did not directly evaluate MDT composition or perioperative processes, and these mechanisms remain speculative within the context of the current analysis. Further research is warranted to determine the extent to which MDT structure and perioperative management influence postoperative outcomes in metabolic bariatric surgery.

### Centralization

Centralization of complex upper gastrointestinal surgery, including anastomotic procedures, has repeatedly been shown to improve patient outcomes. Large-scale healthcare reforms in Ireland, Sweden, and the Netherlands have demonstrated that consolidating care into high-volume centres reduces postoperative mortality and serious complications, likely through enhanced multidisciplinary coordination and procedural expertise [36, 37]. These findings should be interpreted within the broader volume–outcome literature, although in our study significant differences were limited to selected outcomes and overall complication rates remained low across all hospital volume groups. This model of centralization mirrors established practices in cancer and transplant services, where higher procedural volumes and specialist teams have long been associated with improved outcomes [29].

Nonetheless, our data alone are not sufficient to advocate for structural reorganization of bariatric services in the UK. The observed associations between hospital volume and outcomes should instead be interpreted as supportive evidence within the broader volume–outcome literature. In this context, although the UK healthcare system’s centralized and publicly funded structure may differ from more fragmented or insurance-driven models, this organization also promotes standardization of care pathways and enhances data completeness within the NBSR. Therefore, our findings are most generalizable to other European or publicly funded healthcare systems with similar referral mechanisms and centralized governance, while caution is warranted when extrapolating to less centralized or privately funded settings. Further studies incorporating surgeon-level data, operative techniques, and MDT factors are needed to better define how these variables interact within centralized healthcare frameworks.

### Strengths and Limitations

This study benefits from the use of the NBSR, a national registry with broad coverage of UK bariatric procedures and standardized clinical data. Unlike MBSAQIP or NIS, it captures both high- and low-volume centres with medium-term follow-up, supporting population-level analysis. However, it is important to note that the NBSR database is limited by the lack of prospectively collected data beyond 2 years, incomplete recording of complications (as fields are only populated when an event occurs), and potential referral or selection bias in higher-volume centres. Consequently, we were unable to evaluate the impact of hospital volume on longer-term outcomes such as weight loss durability, nutritional status, diabetes remission, or quality of life, which may also be influenced by differences in multidisciplinary follow-up and support. High-volume centres may receive disproportionately complex or higher-risk patients due to their perceived expertise, which could attenuate observable volume–outcome differences. Additionally, variability in data entry practices, coding accuracy, and definitions across institutions may introduce bias. Smaller centres may also have reduced data completeness due to staffing or time constraints. A further limitation is that the NBSR does not capture detailed technical aspects of RYGB construction, including anastomotic technique, jejunojejunostomy configuration, or mesenteric defect closure. Consequently, it was not possible to determine whether variation in operative technique between centres contributed to the observed differences in obstruction rates.

ASA-stratified obstruction rates and procedure selection by ASA category were not prespecified analyses. Further post hoc stratification of the 133 obstruction events could produce unstable estimates and would not resolve confounding by clinical selection. Residual confounding from the interaction between patient risk, procedure choice, centre volume, and referral patterns therefore remains possible.

Furthermore, categorizing hospital volume into quartiles—although an established and widely used approach in volume–outcome studies such as those by Markar et al.—may obscure potential dose–response relationships and introduce arbitrary thresholds. Another limitation relates to operative technique, as the NBSR does not capture information on closure of mesenteric defects or the type of closure performed. Practice variability in this aspect may contribute to heterogeneity in postoperative obstruction risk, independent of hospital volume. Finally, our analysis focused solely on hospital volume; individual surgeon volume, which has been identified as an independent predictor of postoperative outcomes, may act as a mediator or confounder of the observed hospital volume–outcome relationship [9, 36].

## Conclusions

In our study, treatment at high-volume centres was associated with a reduced risk of postoperative obstruction and Clavien-Dindo grade ≥ III following RYGB, with hospital volume emerging as an independent predictor of this complication. While these findings suggest a potential benefit of centralizing complex bariatric procedures such as RYGB, they should be interpreted in the context of generally low complication rates across all volume settings. Further research is needed to clarify the impact of individual surgeon volume, as well as the potential relationship between surgical volume and long-term weight loss outcomes.

## Supplementary Information

Below is the link to the electronic supplementary material.

Supplementary Material 1

## Data Availability

Data relevant to the study are included in the article or uploaded as supplementary information. Raw data are available upon request.

## Declarations

### Transparency

The lead author affirms that this manuscript is an honest, accurate, and transparent account of the study being reported; that no important aspects of the study have been omitted; and that any discrepancies from the study as planned have been explained. All claims expressed in this article are solely those of the authors and do not necessarily represent those of their affiliated organizations.
