# Supplementary material for: The Impact of Hospital Volume on Bariatric Surgery Safety: A Population-Based Study using Data from the National Bariatric Surgery Registry of the United Kingdom
Source: Obes Surg. 2026 Sep 22;36(10):5027–36. doi: 10.1007/s11695-026-08946-1 (PMC13645993; doi:10.1007/s11695-026-08946-1)
Supplement: Supplementary file 1 [file 11695_2026_8946_MOESM1_ESM.docx]

Table S1. Complication Rates in Primary OAGB Patients by Hospital Volume Quartile

| **Characteristic** | **Overall**  N = 4,744^1^ | **I**  N = 817^1^ | **II**  N = 1,175^1^ | **III**  N = 1,317^1^ | **IV**  N = 1,435^1^ | **p-value**^2^ |  |
| --- | --- | --- | --- | --- | --- | --- | --- |
| **Leak** | 9 (0.2%) | 2 (0.2%) | 4 (0.3%) | 1 (<0.1%) | 2 (0.1%) | 0.444 |  |
| **Bleeding** | 14 (0.3%) | 1 (0.1%) | 7 (0.6%) | 3 (0.2%) | 3 (0.2%) | 0.247 |  |
| **Obstruction** | 6 (0.1%) | 1 (0.1%) | 1 (<0.1%) | 2 (0.2%) | 2 (0.1%) | >0.9 |  |
| **CD3+** | 11 (0.2%) | 1 (0.1%) | 4 (0.3%) | 3 (0.2%) | 3 (0.2%) | 0.852 |  |
| ^1^n (%) | | | | | | | |
| ^2^Pearson's Chi-squared test; Fisher's exact test | | | | | | | |

Table S2. Causes of obstruction in RYGB stratified by hospital volume quartile

| **Cause of Obstruction** | **Quartile I** | **Quartile II** | **Quartile III** | **Quartile IV** | **Total** |
| --- | --- | --- | --- | --- | --- |
| **Adhesions** | 5 | 2 | 4 | 0 | 11 |
| **Anastomotic anatomy** | 8 | 12 | 8 | 7 | 35 |
| **Mesenteric anastomosis defect** | 3 | 2 | 1 | 0 | 6 |
| **Internal Hernia** | 7 | 6 | 3 | 0 | 16 |
| **Other (unspecified)** | 12 | 8 | 12 | 4 | 36 |

Obstruction was defined according to NBSR registry coding. Where available, obstruction events were subclassified as adhesions, anastomotic anatomy-related obstruction, mesenteric anastomosis defect, internal hernia, or other causes. Detailed cause data were not recorded for all obstruction events.

Table S3. Logistic Regression: Predictors of Obstruction after Primary RYGB

| **Characteristic** | **OR** | **95% CI** | **p-value** |
| --- | --- | --- | --- |
| **Hospital volume quartile** |  |  |  |
| I | — | — |  |
| II | 0.93 | 0.58 1.49 | 0.8 |
| III | 0.90 | 0.56 1.46 | 0.7 |
| IV | 0.31 | 0.16 0.58 | <0.001 |
| **Age** | 1.0 | 0.98 1.01 | 0.6 |
| **Gender** |  |  |  |
| Female | — | — |  |
| Male | 1.15 | 0.69 1.84 | 0.6 |
| **Type 2 Diabetes** |  |  |  |
| No | — | — |  |
| Yes | 0.71 | 0.44 1.10 | 0.14 |
| **BMI** | 0.99 | 0.97 1.00 | 0.5 |
| **Hypertension** |  |  |  |
| Hypertension on treatment | — | — |  |
| No indication of hypertension; or on no treatment | 1.04 | 0.67 1.63 | 0.9 |
| **Cardiovascular Disease** |  |  |  |
| Diagnosed atherosclerosis | — | — |  |
| No indication of atherosclerosis | 0.43 | 0.23 0.92 | 0.018 |
| **Sleep Apnoea** |  |  |  |
| No | — | — |  |
| Yes - treated | 0.68 | 0.40 1.11 | 0.14 |
| Yes - untreated | 0.27 | 0.02 1.20 | 0.2 |
| **Functional Status** |  |  |  |
| Can climb 1 flight of stairs without resting | — | — |  |
| Can climb 3 flights of stairs without resting | 0.91 | 0.60 1.35 | 0.6 |
| Can climb half a flight of stairs without resting | 1.12 | 0.61 1.94 | 0.7 |
| Requires wheelchair / house bound | 1.50 | 0.36 4.13 | 0.5 |
| **Funding Source** |  |  |  |
| NHS | — | — |  |
| Private | 1.23 | 0.74 1.96 | 0.4 |
| Abbreviations: CI = Confidence Interval OR = Odds Ratio | | | |

Table S4. Logistic Regression: Predictors of Leak after Primary RYGB

| **Characteristic** | **OR** | **95% CI** | **p-value** |
| --- | --- | --- | --- |
| **Hospital volume quartile** |  |  |  |
| I | — | — |  |
| II | 0.85 | 0.42 1.72 | 0.6 |
| III | 0.98 | 0.51 1.95 | >0.9 |
| IV | 1.02 | 0.54 1.99 | >0.9 |
| **Age** | 0.99 | 0.97 1.01 | 0.5 |
| **Gender** |  |  |  |
| Female | — | — |  |
| Male | 1.04 | 0.56 1.83 | 0.9 |
| **Type 2 Diabetes** |  |  |  |
| No | — | — |  |
| Yes | 0.84 | 0.49 1.40 | 0.5 |
| **BMI** | 1.00 |  | >0.9 |
| **Hypertension** |  |  |  |
| Hypertension on treatment | — | — |  |
| No indication of hypertension; or on no treatment | 0.68 | 0.40 1.16 | 0.2 |
| **Cardiovascular Disease** |  |  |  |
| Diagnosed atherosclerosis | — | — |  |
| No indication of atherosclerosis | 0.61 | 0.27 1.63 | 0.3 |
| **Sleep Apnoea** |  |  |  |
| No | — | — |  |
| Yes - treated | 1.11 | 0.63 1.89 | 0.7 |
| Yes - untreated | 0.50 | 0.03 2.28 | 0.5 |
| **Functional Status** |  |  |  |
| Can climb 1 flight of stairs without resting | — | — |  |
| Can climb 3 flights of stairs without resting | 1.22 | 0.73 2.02 | 0.4 |
| Can climb half a flight of stairs without resting | 1.18 | 0.55 2.32 | 0.6 |
| Requires wheelchair / house bound | 1.42 | 0.23 4.74 | 0.6 |
| **Funding Source** |  |  |  |
| NHS | — | — |  |
| Private | 0.59 | 0.22 1.29 | 0.2 |
| Abbreviations: CI = Confidence Interval OR = Odds Ratio | | | |

Table S5. Logistic Regression: Predictors of CD3+ Complications after Primary RYGB

| **Characteristic** | **OR** | **95% CI** | **p-value** |
| --- | --- | --- | --- |
| **Hospital volume quartile** |  |  |  |
| I | — | — |  |
| II | 0.98 | 0.70 1.35 | 0.8 |
| III | 0.94 | 0.68 1.30 | 0.7 |
| IV | 0.71 | 0.51 0.99 | 0.044 |
| **Age** | 1.00 | 0.99 1.01 | 0.6 |
| **Gender** |  |  |  |
| Female | — | — |  |
| Male | 0.82 | 0.58 1.12 | 0.2 |
| **Type 2 Diabetes** |  |  |  |
| No | — | — |  |
| Yes | 0.78 | 0.60 1.02 | 0.074 |
| **BMI** | 1.00 |  | >0.9 |
| **Hypertension** |  |  |  |
| Hypertension on treatment | — | — |  |
| No indication of hypertension; or on no treatment | 0.89 | 0.69 1.17 | 0.4 |
| **Cardiovascular Disease** |  |  |  |
| Diagnosed atherosclerosis | — | — |  |
| No indication of atherosclerosis | 0.58 | 0.38 0.92 | 0.015 |
| **Sleep Apnoea** |  |  |  |
| No | — | — |  |
| Yes - treated | 0.80 | 0.59 1.07 | 0.14 |
| Yes - untreated | 0.53 | 0.19 1.16 | 0.2 |
| **Functional Status** |  |  |  |
| Can climb 1 flight of stairs without resting | — | — |  |
| Can climb 3 flights of stairs without resting | 0.94 | 0.72 1.21 | 0.6 |
| Can climb half a flight of stairs without resting | 1.33 | 0.95 1.84 | 0.085 |
| Requires wheelchair / house bound | 1.53 | 0.72 2.86 | 0.2 |
| **Funding Source** |  |  |  |
| NHS | — | — |  |
| Private | 1.00 | 0.70 1.38 | >0.9 |
| Abbreviations: CI = Confidence Interval OR = Odds Ratio | | | |

Table S6. Logistic Regression: Predictors of Leak after Primary SG

| **Characteristic** | **OR** | **95% CI** | **p-value** |
| --- | --- | --- | --- |
| **Hospital volume quartile** |  |  |  |
| I | — | — |  |
| II | 0.68 | 0.17 2.39 | 0.5 |
| III | 0.65 | 0.16 2.33 | 0.5 |
| IV | 0.37 | 0.05 1.63 | 0.2 |
| **Age** | 1.06 | 1.00 1.11 | 0.038 |
| **Gender** |  |  |  |
| Female | — | — |  |
| Male | 0.79 | 0.17 2.61 | 0.7 |
| **Type 2 Diabetes** |  |  |  |
| No | — | — |  |
| Yes | 0.86 | 0.23 2.70 | 0.8 |
| **BMI** | 0.97 | 0.91 1.02 | 0.4 |
| **Hypertension** |  |  |  |
| Hypertension on treatment | — | — |  |
| No indication of hypertension; or on no treatment | 1.54 | 0.49 5.40 | 0.5 |
| **Cardiovascular Disease** |  |  |  |
| Diagnosed atherosclerosis | — | — |  |
| No indication of atherosclerosis | 6379918 | 0.00 NA | >0.9 |
| **Sleep Apnoea** |  |  |  |
| No | — | — |  |
| Yes - treated | 1.66 | 0.48 5.06 | 0.4 |
| Yes - untreated | 0.00 |  | >0.9 |
| **Functional Status** |  |  |  |
| Can climb 1 flight of stairs without resting | — | — |  |
| Can climb 3 flights of stairs without resting | 1.04 | 0.30 3.38 | >0.9 |
| Can climb half a flight of stairs without resting | 1.41 | 0.37 4.76 | 0.6 |
| Requires wheelchair / house bound | 0.00 |  | >0.9 |
| **Funding Source** |  |  |  |
| NHS | — | — |  |
| Private | 0.38 | 0.08 1.31 | 0.2 |
| Abbreviations: CI = Confidence Interval OR = Odds Ratio | | | |

Table S7. Logistic Regression: Predictors of CD3+ after Primary SG

| **Characteristic** | **OR** | **95% CI** | **p-value** |
| --- | --- | --- | --- |
| **Hospital volume quartile** |  |  |  |
| I | — | — |  |
| II | 0.64 | 0.13 2.62 | 0.5 |
| III | 1.55 | 0.51 5.26 | 0.4 |
| IV | 0.46 | 0.07 2.19 | 0.4 |
| **Age** | 1.01 | 0.96 1.06 | 0.7 |
| **Gender** |  |  |  |
| Female | — | — |  |
| Male | 1.04 | 0.28 3.08 | >0.9 |
| **Type 2 Diabetes** |  |  |  |
| No | — | — |  |
| Yes | 1.08 | 0.32 3.17 | 0.9 |
| **BMI** | 0.95 | 0.90 1.00 | 0.14 |
| **Hypertension** |  |  |  |
| Hypertension on treatment | — | — |  |
| No indication of hypertension; or on no treatment | 1.03 | 0.35 3.31 | >0.9 |
| **Cardiovascular Disease** |  |  |  |
| Diagnosed atherosclerosis | — | — |  |
| No indication of atherosclerosis | 5451993 | 0.00 NA | >0.9 |
| **Sleep Apnoea** |  |  |  |
| No | — | — |  |
| Yes - treated | 1.54 | 0.45 4.61 | 0.5 |
| Yes - untreated | 0.00 |  | >0.9 |
| **Functional Status** |  |  |  |
| Can climb 1 flight of stairs without resting | — | — |  |
| Can climb 3 flights of stairs without resting | 0.36 | 0.10 1.06 | 0.084 |
| Can climb half a flight of stairs without resting | 0.23 | 0.01 1.15 | 0.2 |
| Requires wheelchair / house bound | 0.00 |  | >0.9 |
| **Funding Source** |  |  |  |
| NHS | — | — |  |
| Private | 0.58 | 0.15 1.81 | 0.4 |
| Abbreviations: CI = Confidence Interval OR = Odds Ratio | | | |

**Table S8.** Hospital procedure counts

| Number and Percentage of Procedures per Hospital (n (%)) | | | | | |
| --- | --- | --- | --- | --- | --- |
| Hospital | RYGB | Sleeve | OAGB | Revisional | Total (n) |
| 0001 | 63 | 85 | 0 | 0 | 148 |
| 0002 | 33 | 331 | 25 | 0 | 389 |
| 0003 | 77 | 130 | 7 | 1 | 215 |
| 0005 | 402 | 85 | 31 | 0 | 518 |
| 0006 | 850 | 247 | 30 | 18 | 1145 |
| 0008 | 300 | 779 | 446 | 6 | 1531 |
| 0015 | 443 | 402 | 5 | 2 | 852 |
| 0023 | 201 | 504 | 0 | 0 | 705 |
| 0029 | 384 | 97 | 0 | 0 | 481 |
| 0032 | 471 | 626 | 247 | 1 | 1345 |
| 0034 | 275 | 120 | 8 | 1 | 404 |
| 0035 | 305 | 123 | 0 | 0 | 428 |
| 0036 | 160 | 209 | 19 | 0 | 388 |
| 0037 | 97 | 20 | 0 | 0 | 117 |
| 0038 | 256 | 77 | 0 | 0 | 333 |
| 0039 | 65 | 341 | 0 | 4 | 410 |
| 0041 | 53 | 261 | 353 | 7 | 674 |
| 0042 | 123 | 467 | 43 | 0 | 633 |
| 0043 | 546 | 329 | 0 | 21 | 896 |
| 0044 | 119 | 59 | 0 | 0 | 178 |
| 0046 | 292 | 190 | 0 | 0 | 482 |
| 0047 | 525 | 831 | 17 | 1 | 1374 |
| 0048 | 388 | 232 | 20 | 0 | 640 |
| 0050 | 274 | 127 | 93 | 0 | 494 |
| 0051 | 49 | 70 | 0 | 0 | 119 |
| 0053 | 150 | 227 | 398 | 7 | 782 |
| 0054 | 175 | 219 | 10 | 0 | 404 |
| 0060 | 712 | 1073 | 270 | 17 | 2072 |
| 0061 | 245 | 289 | 102 | 0 | 636 |
| 0062 | 1057 | 1170 | 191 | 2 | 2420 |
| 0063 | 593 | 50 | 194 | 2 | 839 |
| 0064 | 565 | 704 | 4 | 18 | 1291 |
| 0067 | 18 | 100 | 3 | 0 | 121 |
| 0069 | 1574 | 806 | 10 | 6 | 2396 |
| 0071 | 127 | 437 | 18 | 0 | 582 |
| 0075 | 172 | 181 | 0 | 4 | 357 |
| 0079 | 77 | 549 | 5 | 3 | 634 |
| 0080 | 28 | 72 | 19 | 0 | 119 |
| 0082 | 33 | 168 | 0 | 0 | 201 |
| 0086 | 616 | 299 | 2 | 21 | 938 |
| 0087 | 57 | 80 | 0 | 0 | 137 |
| 0091 | 50 | 141 | 0 | 0 | 191 |
| 0092 | 113 | 191 | 18 | 1 | 323 |
| 0095 | 88 | 177 | 1 | 7 | 273 |
| 0097 | 420 | 196 | 0 | 27 | 643 |
| 0106 | 1635 | 325 | 22 | 0 | 1982 |
| 0107 | 395 | 245 | 26 | 4 | 670 |
| 0120 | 42 | 87 | 539 | 7 | 675 |
| 0122 | 698 | 640 | 98 | 1 | 1437 |
| 0125 | 183 | 219 | 0 | 0 | 402 |
| 0126 | 614 | 313 | 3 | 0 | 930 |
| 0127 | 564 | 191 | 0 | 2 | 757 |
| 0128 | 6 | 179 | 0 | 0 | 185 |
| 0129 | 58 | 60 | 0 | 0 | 118 |
| 0130 | 70 | 209 | 17 | 0 | 296 |
| 0132 | 24 | 88 | 2 | 0 | 114 |
| 0134 | 485 | 334 | 1 | 0 | 820 |
| 0137 | 333 | 169 | 291 | 8 | 801 |
| 0140 | 2 | 135 | 3 | 0 | 140 |
| 0141 | 220 | 175 | 0 | 0 | 395 |
| 0143 | 149 | 228 | 0 | 0 | 377 |
| 0146 | 280 | 457 | 0 | 1 | 738 |
| 0148 | 38 | 73 | 1 | 0 | 112 |
| 0149 | 112 | 1031 | 48 | 0 | 1191 |
| 0150 | 11 | 333 | 0 | 0 | 344 |
| 0153 | 68 | 75 | 0 | 0 | 143 |
| 0156 | 60 | 59 | 0 | 0 | 119 |
| 0157 | 500 | 270 | 0 | 0 | 770 |
| 0159 | 69 | 229 | 1 | 0 | 299 |
| 0161 | 1 | 142 | 1 | 2 | 146 |
| 0164 | 90 | 190 | 41 | 0 | 321 |
| 0165 | 2 | 156 | 0 | 0 | 158 |
| 0167 | 1611 | 639 | 16 | 2 | 2268 |
| 0170 | 521 | 983 | 510 | 10 | 2024 |
| 0171 | 487 | 557 | 25 | 4 | 1073 |
| 0172 | 126 | 100 | 31 | 0 | 257 |
| 0174 | 1057 | 189 | 435 | 3 | 1684 |
| 0176 | 94 | 192 | 147 | 0 | 433 |
| 0177 | 71 | 71 | 42 | 0 | 184 |
| 0182 | 574 | 46 | 24 | 1 | 645 |
| 0188 | 343 | 827 | 48 | 16 | 1234 |
| 0189 | 449 | 92 | 51 | 2 | 594 |
| 0190 | 25 | 558 | 0 | 6 | 589 |
| 0191 | 64 | 70 | 0 | 1 | 135 |
| 0192 | 188 | 364 | 62 | 1 | 615 |
| 0195 | 1 | 222 | 3 | 1 | 227 |
| 0196 | 278 | 290 | 0 | 2 | 570 |
| 0197 | 486 | 431 | 229 | 1 | 1147 |
| 0198 | 137 | 34 | 0 | 0 | 171 |

**Table S9.** Annual and moving average volume per hospital/year

| Hospital | Year | Annual Volume | Moving Average Volume |
| --- | --- | --- | --- |
| 0001 | 2,016 | 22 | 22.0 |
| 0001 | 2,017 | 31 | 26.5 |
| 0001 | 2,018 | 32 | 28.3 |
| 0001 | 2,019 | 40 | 31.2 |
| 0001 | 2,020 | 17 | 30.0 |
| 0001 | 2,023 | 53 | 35.5 |
| 0002 | 2,018 | 15 | 15.0 |
| 0002 | 2,019 | 40 | 27.5 |
| 0002 | 2,020 | 22 | 25.7 |
| 0002 | 2,021 | 83 | 40.0 |
| 0002 | 2,022 | 163 | 77.0 |
| 0002 | 2,023 | 77 | 86.2 |
| 0003 | 2,014 | 12 | 12.0 |
| 0003 | 2,015 | 19 | 15.5 |
| 0003 | 2,016 | 37 | 22.7 |
| 0003 | 2,017 | 45 | 28.2 |
| 0003 | 2,018 | 41 | 35.5 |
| 0003 | 2,019 | 34 | 39.2 |
| 0003 | 2,020 | 11 | 32.8 |
| 0003 | 2,021 | 35 | 30.2 |
| 0003 | 2,022 | 26 | 26.5 |
| 0004 | 2,015 | 13 | 13.0 |
| 0004 | 2,016 | 28 | 20.5 |
| 0004 | 2,017 | 19 | 20.0 |
| 0004 | 2,019 | 10 | 17.5 |
| 0005 | 2,020 | 94 | 94.0 |
| 0005 | 2,021 | 159 | 126.5 |
| 0005 | 2,022 | 210 | 154.3 |
| 0005 | 2,023 | 54 | 129.2 |
| 0006 | 2,014 | 95 | 95.0 |
| 0006 | 2,015 | 151 | 123.0 |
| 0006 | 2,016 | 174 | 140.0 |
| 0006 | 2,017 | 180 | 150.0 |
| 0006 | 2,018 | 278 | 195.8 |
| 0006 | 2,019 | 244 | 219.0 |
| 0006 | 2,020 | 43 | 186.2 |
| 0006 | 2,021 | 48 | 153.2 |
| 0006 | 2,022 | 76 | 102.8 |
| 0006 | 2,023 | 28 | 48.8 |
| 0008 | 2,014 | 13 | 13.0 |
| 0008 | 2,015 | 11 | 12.0 |
| 0008 | 2,016 | 33 | 19.0 |
| 0008 | 2,017 | 126 | 45.8 |
| 0008 | 2,018 | 155 | 81.2 |
| 0008 | 2,019 | 173 | 121.8 |
| 0008 | 2,020 | 132 | 146.5 |
| 0008 | 2,021 | 441 | 225.2 |
| 0008 | 2,022 | 390 | 284.0 |
| 0008 | 2,023 | 167 | 282.5 |
| 0010 | 2,021 | 10 | 10.0 |
| 0013 | 2,022 | 27 | 27.0 |
| 0013 | 2,023 | 51 | 39.0 |
| 0015 | 2,014 | 157 | 157.0 |
| 0015 | 2,015 | 168 | 162.5 |
| 0015 | 2,016 | 195 | 173.3 |
| 0015 | 2,017 | 223 | 185.8 |
| 0015 | 2,018 | 190 | 194.0 |
| 0015 | 2,019 | 161 | 192.2 |
| 0015 | 2,020 | 44 | 154.5 |
| 0015 | 2,022 | 41 | 109.0 |
| 0016 | 2,020 | 12 | 12.0 |
| 0017 | 2,022 | 87 | 87.0 |
| 0021 | 2,018 | 21 | 21.0 |
| 0021 | 2,019 | 20 | 20.5 |
| 0021 | 2,020 | 27 | 22.7 |
| 0021 | 2,021 | 49 | 29.2 |
| 0021 | 2,022 | 25 | 30.2 |
| 0021 | 2,023 | 13 | 28.5 |
| 0023 | 2,014 | 80 | 80.0 |
| 0023 | 2,015 | 132 | 106.0 |
| 0023 | 2,016 | 105 | 105.7 |
| 0023 | 2,017 | 84 | 100.2 |
| 0023 | 2,018 | 65 | 96.5 |
| 0023 | 2,019 | 96 | 87.5 |
| 0023 | 2,020 | 52 | 74.2 |
| 0023 | 2,021 | 70 | 70.8 |
| 0023 | 2,022 | 42 | 65.0 |
| 0023 | 2,023 | 17 | 45.2 |
| 0024 | 2,017 | 11 | 11.0 |
| 0024 | 2,018 | 12 | 11.5 |
| 0025 | 2,020 | 10 | 10.0 |
| 0026 | 2,018 | 10 | 10.0 |
| 0027 | 2,014 | 10 | 10.0 |
| 0028 | 2,020 | 12 | 12.0 |
| 0028 | 2,021 | 25 | 18.5 |
| 0029 | 2,014 | 54 | 54.0 |
| 0029 | 2,015 | 75 | 64.5 |
| 0029 | 2,016 | 75 | 68.0 |
| 0029 | 2,017 | 66 | 67.5 |
| 0029 | 2,018 | 37 | 63.2 |
| 0029 | 2,019 | 25 | 50.8 |
| 0029 | 2,021 | 29 | 39.2 |
| 0029 | 2,022 | 77 | 42.0 |
| 0029 | 2,023 | 34 | 41.2 |
| 0030 | 2,014 | 34 | 34.0 |
| 0030 | 2,016 | 11 | 22.5 |
| 0030 | 2,017 | 18 | 21.0 |
| 0030 | 2,019 | 18 | 20.2 |
| 0032 | 2,014 | 62 | 62.0 |
| 0032 | 2,015 | 64 | 63.0 |
| 0032 | 2,016 | 37 | 54.3 |
| 0032 | 2,017 | 46 | 52.2 |
| 0032 | 2,018 | 83 | 57.5 |
| 0032 | 2,019 | 143 | 77.2 |
| 0032 | 2,020 | 234 | 126.5 |
| 0032 | 2,021 | 337 | 199.2 |
| 0032 | 2,022 | 326 | 260.0 |
| 0032 | 2,023 | 140 | 259.2 |
| 0034 | 2,014 | 209 | 209.0 |
| 0034 | 2,015 | 236 | 222.5 |
| 0034 | 2,016 | 30 | 158.3 |
| 0035 | 2,014 | 62 | 62.0 |
| 0035 | 2,015 | 58 | 60.0 |
| 0035 | 2,016 | 80 | 66.7 |
| 0035 | 2,017 | 46 | 61.5 |
| 0035 | 2,018 | 50 | 58.5 |
| 0035 | 2,019 | 80 | 64.0 |
| 0035 | 2,020 | 26 | 50.5 |
| 0035 | 2,021 | 16 | 43.0 |
| 0035 | 2,022 | 28 | 37.5 |
| 0035 | 2,023 | 13 | 20.8 |
| 0036 | 2,014 | 61 | 61.0 |
| 0036 | 2,015 | 80 | 70.5 |
| 0036 | 2,016 | 59 | 66.7 |
| 0036 | 2,017 | 79 | 69.8 |
| 0036 | 2,018 | 55 | 68.2 |
| 0036 | 2,019 | 49 | 60.5 |
| 0036 | 2,021 | 14 | 49.2 |
| 0036 | 2,022 | 19 | 34.2 |
| 0037 | 2,022 | 51 | 51.0 |
| 0037 | 2,023 | 69 | 60.0 |
| 0038 | 2,014 | 21 | 21.0 |
| 0038 | 2,015 | 49 | 35.0 |
| 0038 | 2,016 | 34 | 34.7 |
| 0038 | 2,017 | 32 | 34.0 |
| 0038 | 2,018 | 33 | 37.0 |
| 0038 | 2,019 | 39 | 34.5 |
| 0038 | 2,021 | 54 | 39.5 |
| 0038 | 2,022 | 103 | 57.2 |
| 0038 | 2,023 | 12 | 52.0 |
| 0039 | 2,014 | 64 | 64.0 |
| 0039 | 2,015 | 60 | 62.0 |
| 0039 | 2,016 | 75 | 66.3 |
| 0039 | 2,017 | 81 | 70.0 |
| 0039 | 2,018 | 57 | 68.2 |
| 0039 | 2,019 | 39 | 63.0 |
| 0039 | 2,021 | 76 | 63.2 |
| 0039 | 2,022 | 31 | 50.8 |
| 0039 | 2,023 | 38 | 46.0 |
| 0041 | 2,014 | 205 | 205.0 |
| 0041 | 2,015 | 152 | 178.5 |
| 0041 | 2,016 | 115 | 157.3 |
| 0041 | 2,017 | 152 | 156.0 |
| 0041 | 2,018 | 146 | 141.2 |
| 0041 | 2,019 | 97 | 127.5 |
| 0041 | 2,020 | 45 | 110.0 |
| 0042 | 2,014 | 30 | 30.0 |
| 0042 | 2,015 | 25 | 27.5 |
| 0042 | 2,016 | 40 | 31.7 |
| 0042 | 2,017 | 55 | 37.5 |
| 0042 | 2,018 | 84 | 51.0 |
| 0042 | 2,019 | 78 | 64.2 |
| 0042 | 2,020 | 65 | 70.5 |
| 0042 | 2,021 | 120 | 86.8 |
| 0042 | 2,022 | 134 | 99.2 |
| 0042 | 2,023 | 28 | 86.8 |
| 0043 | 2,014 | 180 | 180.0 |
| 0043 | 2,015 | 192 | 186.0 |
| 0043 | 2,016 | 183 | 185.0 |
| 0043 | 2,017 | 181 | 184.0 |
| 0043 | 2,018 | 131 | 171.8 |
| 0043 | 2,019 | 105 | 150.0 |
| 0043 | 2,020 | 44 | 115.2 |
| 0043 | 2,021 | 52 | 83.0 |
| 0043 | 2,022 | 94 | 73.8 |
| 0043 | 2,023 | 56 | 61.5 |
| 0044 | 2,014 | 26 | 26.0 |
| 0044 | 2,015 | 32 | 29.0 |
| 0044 | 2,016 | 44 | 34.0 |
| 0044 | 2,017 | 42 | 36.0 |
| 0044 | 2,018 | 20 | 34.5 |
| 0044 | 2,019 | 22 | 32.0 |
| 0045 | 2,014 | 18 | 18.0 |
| 0045 | 2,015 | 12 | 15.0 |
| 0045 | 2,016 | 24 | 18.0 |
| 0045 | 2,017 | 21 | 18.8 |
| 0045 | 2,018 | 10 | 16.8 |
| 0046 | 2,014 | 64 | 64.0 |
| 0046 | 2,015 | 34 | 49.0 |
| 0046 | 2,016 | 51 | 49.7 |
| 0046 | 2,017 | 64 | 53.2 |
| 0046 | 2,018 | 46 | 48.8 |
| 0046 | 2,019 | 43 | 51.0 |
| 0046 | 2,020 | 51 | 51.0 |
| 0046 | 2,021 | 48 | 47.0 |
| 0046 | 2,022 | 62 | 51.0 |
| 0046 | 2,023 | 26 | 46.8 |
| 0047 | 2,014 | 112 | 112.0 |
| 0047 | 2,015 | 143 | 127.5 |
| 0047 | 2,016 | 189 | 148.0 |
| 0047 | 2,017 | 283 | 181.8 |
| 0047 | 2,018 | 199 | 203.5 |
| 0047 | 2,019 | 263 | 233.5 |
| 0047 | 2,020 | 157 | 225.5 |
| 0047 | 2,021 | 81 | 175.0 |
| 0047 | 2,022 | 136 | 159.2 |
| 0047 | 2,023 | 73 | 111.8 |
| 0048 | 2,014 | 128 | 128.0 |
| 0048 | 2,015 | 80 | 104.0 |
| 0048 | 2,016 | 64 | 90.7 |
| 0048 | 2,017 | 75 | 86.8 |
| 0048 | 2,018 | 83 | 75.5 |
| 0048 | 2,019 | 74 | 74.0 |
| 0048 | 2,020 | 25 | 64.2 |
| 0048 | 2,021 | 44 | 56.5 |
| 0048 | 2,022 | 37 | 45.0 |
| 0048 | 2,023 | 58 | 41.0 |
| 0049 | 2,017 | 77 | 77.0 |
| 0049 | 2,018 | 61 | 69.0 |
| 0049 | 2,019 | 51 | 63.0 |
| 0050 | 2,014 | 76 | 76.0 |
| 0050 | 2,015 | 43 | 59.5 |
| 0050 | 2,016 | 90 | 69.7 |
| 0050 | 2,017 | 74 | 70.8 |
| 0050 | 2,018 | 61 | 67.0 |
| 0050 | 2,019 | 89 | 78.5 |
| 0050 | 2,020 | 19 | 60.8 |
| 0050 | 2,021 | 18 | 46.8 |
| 0050 | 2,022 | 27 | 38.2 |
| 0050 | 2,023 | 13 | 19.2 |
| 0051 | 2,020 | 24 | 24.0 |
| 0051 | 2,021 | 23 | 23.5 |
| 0051 | 2,022 | 25 | 24.0 |
| 0051 | 2,023 | 16 | 22.0 |
| 0053 | 2,016 | 48 | 48.0 |
| 0053 | 2,017 | 65 | 56.5 |
| 0053 | 2,018 | 140 | 84.3 |
| 0053 | 2,019 | 298 | 137.8 |
| 0053 | 2,020 | 129 | 158.0 |
| 0053 | 2,021 | 175 | 185.5 |
| 0053 | 2,022 | 177 | 194.8 |
| 0053 | 2,023 | 27 | 127.0 |
| 0054 | 2,014 | 53 | 53.0 |
| 0054 | 2,015 | 61 | 57.0 |
| 0054 | 2,016 | 72 | 62.0 |
| 0054 | 2,017 | 56 | 60.5 |
| 0054 | 2,018 | 43 | 58.0 |
| 0054 | 2,019 | 20 | 47.8 |
| 0054 | 2,020 | 17 | 34.0 |
| 0054 | 2,021 | 27 | 26.8 |
| 0054 | 2,022 | 51 | 28.8 |
| 0054 | 2,023 | 36 | 32.8 |
| 0058 | 2,014 | 16 | 16.0 |
| 0058 | 2,015 | 23 | 19.5 |
| 0058 | 2,016 | 23 | 20.7 |
| 0059 | 2,020 | 10 | 10.0 |
| 0060 | 2,014 | 306 | 306.0 |
| 0060 | 2,015 | 347 | 326.5 |
| 0060 | 2,016 | 209 | 287.3 |
| 0060 | 2,017 | 318 | 295.0 |
| 0060 | 2,018 | 220 | 273.5 |
| 0060 | 2,019 | 248 | 248.8 |
| 0060 | 2,020 | 126 | 228.0 |
| 0060 | 2,021 | 181 | 193.8 |
| 0060 | 2,022 | 127 | 170.5 |
| 0060 | 2,023 | 89 | 130.8 |
| 0061 | 2,014 | 138 | 138.0 |
| 0061 | 2,015 | 120 | 129.0 |
| 0061 | 2,016 | 107 | 121.7 |
| 0061 | 2,017 | 22 | 96.8 |
| 0061 | 2,018 | 34 | 70.8 |
| 0061 | 2,019 | 55 | 54.5 |
| 0061 | 2,020 | 18 | 32.2 |
| 0061 | 2,021 | 29 | 34.0 |
| 0061 | 2,022 | 90 | 48.0 |
| 0061 | 2,023 | 40 | 44.2 |
| 0062 | 2,014 | 327 | 327.0 |
| 0062 | 2,015 | 312 | 319.5 |
| 0062 | 2,016 | 292 | 310.3 |
| 0062 | 2,017 | 282 | 303.2 |
| 0062 | 2,018 | 233 | 279.8 |
| 0062 | 2,019 | 311 | 279.5 |
| 0062 | 2,020 | 222 | 262.0 |
| 0062 | 2,021 | 242 | 252.0 |
| 0062 | 2,022 | 251 | 256.5 |
| 0062 | 2,023 | 135 | 212.5 |
| 0063 | 2,014 | 159 | 159.0 |
| 0063 | 2,015 | 145 | 152.0 |
| 0063 | 2,016 | 151 | 151.7 |
| 0063 | 2,017 | 139 | 148.5 |
| 0063 | 2,018 | 130 | 141.2 |
| 0063 | 2,019 | 74 | 123.5 |
| 0063 | 2,021 | 35 | 94.5 |
| 0063 | 2,022 | 25 | 66.0 |
| 0063 | 2,023 | 22 | 39.0 |
| 0064 | 2,014 | 71 | 71.0 |
| 0064 | 2,015 | 172 | 121.5 |
| 0064 | 2,016 | 196 | 146.3 |
| 0064 | 2,017 | 238 | 169.2 |
| 0064 | 2,018 | 394 | 250.0 |
| 0064 | 2,019 | 349 | 294.2 |
| 0064 | 2,021 | 13 | 248.5 |
| 0066 | 2,018 | 16 | 16.0 |
| 0066 | 2,020 | 11 | 13.5 |
| 0066 | 2,021 | 14 | 13.7 |
| 0066 | 2,022 | 15 | 14.0 |
| 0067 | 2,015 | 25 | 25.0 |
| 0067 | 2,016 | 57 | 41.0 |
| 0067 | 2,017 | 38 | 40.0 |
| 0067 | 2,018 | 16 | 34.0 |
| 0069 | 2,014 | 170 | 170.0 |
| 0069 | 2,015 | 211 | 190.5 |
| 0069 | 2,016 | 327 | 236.0 |
| 0069 | 2,017 | 510 | 304.5 |
| 0069 | 2,018 | 397 | 361.2 |
| 0069 | 2,019 | 341 | 393.8 |
| 0069 | 2,020 | 135 | 345.8 |
| 0069 | 2,021 | 165 | 259.5 |
| 0069 | 2,022 | 111 | 188.0 |
| 0069 | 2,023 | 82 | 123.2 |
| 0071 | 2,014 | 27 | 27.0 |
| 0071 | 2,015 | 60 | 43.5 |
| 0071 | 2,016 | 66 | 51.0 |
| 0071 | 2,017 | 66 | 54.8 |
| 0071 | 2,018 | 73 | 66.2 |
| 0071 | 2,019 | 78 | 70.8 |
| 0071 | 2,020 | 30 | 61.8 |
| 0071 | 2,021 | 87 | 67.0 |
| 0071 | 2,022 | 67 | 65.5 |
| 0071 | 2,023 | 33 | 54.2 |
| 0072 | 2,015 | 11 | 11.0 |
| 0075 | 2,014 | 100 | 100.0 |
| 0075 | 2,015 | 72 | 86.0 |
| 0075 | 2,016 | 106 | 92.7 |
| 0075 | 2,017 | 84 | 90.5 |
| 0075 | 2,018 | 45 | 76.8 |
| 0075 | 2,019 | 22 | 64.2 |
| 0075 | 2,021 | 50 | 50.2 |
| 0075 | 2,022 | 28 | 36.2 |
| 0076 | 2,017 | 15 | 15.0 |
| 0076 | 2,018 | 12 | 13.5 |
| 0079 | 2,014 | 21 | 21.0 |
| 0079 | 2,015 | 37 | 29.0 |
| 0079 | 2,016 | 52 | 36.7 |
| 0079 | 2,017 | 109 | 54.8 |
| 0079 | 2,018 | 177 | 93.8 |
| 0079 | 2,019 | 153 | 122.8 |
| 0079 | 2,020 | 99 | 134.5 |
| 0079 | 2,021 | 175 | 151.0 |
| 0079 | 2,022 | 145 | 143.0 |
| 0079 | 2,023 | 26 | 111.2 |
| 0080 | 2,016 | 22 | 22.0 |
| 0080 | 2,017 | 12 | 17.0 |
| 0080 | 2,021 | 55 | 29.7 |
| 0080 | 2,022 | 15 | 26.0 |
| 0081 | 2,022 | 15 | 15.0 |
| 0082 | 2,014 | 31 | 31.0 |
| 0082 | 2,015 | 19 | 25.0 |
| 0082 | 2,016 | 49 | 33.0 |
| 0082 | 2,017 | 71 | 42.5 |
| 0082 | 2,018 | 32 | 42.8 |
| 0085 | 2,015 | 14 | 14.0 |
| 0085 | 2,016 | 13 | 13.5 |
| 0085 | 2,017 | 10 | 12.3 |
| 0086 | 2,014 | 178 | 178.0 |
| 0086 | 2,015 | 190 | 184.0 |
| 0086 | 2,016 | 181 | 183.0 |
| 0086 | 2,017 | 175 | 181.0 |
| 0086 | 2,018 | 119 | 166.2 |
| 0086 | 2,019 | 134 | 152.2 |
| 0086 | 2,020 | 58 | 121.5 |
| 0086 | 2,021 | 29 | 85.0 |
| 0086 | 2,022 | 91 | 78.0 |
| 0086 | 2,023 | 46 | 56.0 |
| 0087 | 2,014 | 15 | 15.0 |
| 0087 | 2,016 | 13 | 14.0 |
| 0087 | 2,017 | 12 | 13.3 |
| 0087 | 2,020 | 23 | 15.8 |
| 0087 | 2,021 | 23 | 17.8 |
| 0087 | 2,022 | 37 | 23.8 |
| 0087 | 2,023 | 28 | 27.8 |
| 0091 | 2,014 | 17 | 17.0 |
| 0091 | 2,015 | 21 | 19.0 |
| 0091 | 2,016 | 26 | 21.3 |
| 0091 | 2,017 | 42 | 26.5 |
| 0091 | 2,018 | 38 | 31.8 |
| 0091 | 2,019 | 26 | 33.0 |
| 0091 | 2,020 | 16 | 30.5 |
| 0092 | 2,014 | 21 | 21.0 |
| 0092 | 2,015 | 25 | 23.0 |
| 0092 | 2,016 | 29 | 25.0 |
| 0092 | 2,017 | 22 | 24.2 |
| 0092 | 2,018 | 30 | 26.5 |
| 0092 | 2,019 | 22 | 25.8 |
| 0092 | 2,020 | 16 | 22.5 |
| 0092 | 2,021 | 55 | 30.8 |
| 0092 | 2,022 | 92 | 46.2 |
| 0092 | 2,023 | 84 | 61.8 |
| 0093 | 2,015 | 11 | 11.0 |
| 0095 | 2,014 | 50 | 50.0 |
| 0095 | 2,015 | 41 | 45.5 |
| 0095 | 2,016 | 62 | 51.0 |
| 0095 | 2,017 | 117 | 67.5 |
| 0095 | 2,018 | 64 | 71.0 |
| 0095 | 2,019 | 20 | 65.8 |
| 0095 | 2,020 | 16 | 54.2 |
| 0095 | 2,021 | 32 | 33.0 |
| 0095 | 2,022 | 19 | 21.8 |
| 0095 | 2,023 | 17 | 21.0 |
| 0096 | 2,018 | 10 | 10.0 |
| 0096 | 2,019 | 26 | 18.0 |
| 0096 | 2,020 | 15 | 17.0 |
| 0096 | 2,021 | 22 | 18.2 |
| 0096 | 2,022 | 41 | 26.0 |
| 0097 | 2,014 | 93 | 93.0 |
| 0097 | 2,015 | 149 | 121.0 |
| 0097 | 2,016 | 102 | 114.7 |
| 0097 | 2,017 | 113 | 114.2 |
| 0097 | 2,018 | 113 | 119.2 |
| 0097 | 2,019 | 105 | 108.2 |
| 0097 | 2,020 | 48 | 94.8 |
| 0097 | 2,021 | 154 | 105.0 |
| 0097 | 2,022 | 143 | 112.5 |
| 0097 | 2,023 | 81 | 106.5 |
| 0099 | 2,018 | 10 | 10.0 |
| 0099 | 2,019 | 22 | 16.0 |
| 0102 | 2,016 | 29 | 29.0 |
| 0102 | 2,017 | 102 | 65.5 |
| 0104 | 2,014 | 18 | 18.0 |
| 0104 | 2,015 | 11 | 14.5 |
| 0106 | 2,014 | 211 | 211.0 |
| 0106 | 2,015 | 258 | 234.5 |
| 0106 | 2,016 | 129 | 199.3 |
| 0106 | 2,017 | 311 | 227.2 |
| 0106 | 2,018 | 333 | 257.8 |
| 0106 | 2,019 | 360 | 283.2 |
| 0106 | 2,020 | 171 | 293.8 |
| 0106 | 2,021 | 119 | 245.8 |
| 0106 | 2,022 | 161 | 202.8 |
| 0106 | 2,023 | 27 | 119.5 |
| 0107 | 2,014 | 109 | 109.0 |
| 0107 | 2,015 | 84 | 96.5 |
| 0107 | 2,016 | 116 | 103.0 |
| 0107 | 2,017 | 66 | 93.8 |
| 0107 | 2,018 | 76 | 85.5 |
| 0107 | 2,019 | 89 | 86.8 |
| 0107 | 2,020 | 20 | 62.8 |
| 0107 | 2,021 | 56 | 60.2 |
| 0107 | 2,022 | 65 | 57.5 |
| 0107 | 2,023 | 46 | 46.8 |
| 0108 | 2,021 | 21 | 21.0 |
| 0108 | 2,022 | 21 | 21.0 |
| 0108 | 2,023 | 27 | 23.0 |
| 0113 | 2,019 | 39 | 39.0 |
| 0117 | 2,015 | 17 | 17.0 |
| 0117 | 2,016 | 15 | 16.0 |
| 0118 | 2,020 | 44 | 44.0 |
| 0118 | 2,021 | 24 | 34.0 |
| 0118 | 2,022 | 22 | 30.0 |
| 0120 | 2,014 | 81 | 81.0 |
| 0120 | 2,015 | 51 | 66.0 |
| 0120 | 2,016 | 51 | 61.0 |
| 0120 | 2,017 | 61 | 61.0 |
| 0120 | 2,018 | 102 | 66.2 |
| 0120 | 2,019 | 91 | 76.2 |
| 0120 | 2,020 | 38 | 73.0 |
| 0120 | 2,021 | 68 | 74.8 |
| 0120 | 2,022 | 83 | 70.0 |
| 0120 | 2,023 | 52 | 60.2 |
| 0122 | 2,014 | 156 | 156.0 |
| 0122 | 2,015 | 165 | 160.5 |
| 0122 | 2,016 | 165 | 162.0 |
| 0122 | 2,017 | 167 | 163.2 |
| 0122 | 2,018 | 173 | 167.5 |
| 0122 | 2,019 | 222 | 181.8 |
| 0122 | 2,020 | 97 | 164.8 |
| 0122 | 2,021 | 109 | 150.2 |
| 0122 | 2,022 | 193 | 155.2 |
| 0122 | 2,023 | 106 | 126.2 |
| 0125 | 2,014 | 50 | 50.0 |
| 0125 | 2,015 | 48 | 49.0 |
| 0125 | 2,016 | 51 | 49.7 |
| 0125 | 2,017 | 52 | 50.2 |
| 0125 | 2,018 | 78 | 57.2 |
| 0125 | 2,019 | 81 | 65.5 |
| 0125 | 2,020 | 28 | 59.8 |
| 0125 | 2,021 | 44 | 57.8 |
| 0125 | 2,022 | 29 | 45.5 |
| 0125 | 2,023 | 28 | 32.2 |
| 0126 | 2,014 | 49 | 49.0 |
| 0126 | 2,015 | 88 | 68.5 |
| 0126 | 2,016 | 96 | 77.7 |
| 0126 | 2,017 | 129 | 90.5 |
| 0126 | 2,018 | 154 | 116.8 |
| 0126 | 2,019 | 153 | 133.0 |
| 0126 | 2,020 | 68 | 126.0 |
| 0126 | 2,021 | 111 | 121.5 |
| 0126 | 2,022 | 101 | 108.2 |
| 0126 | 2,023 | 44 | 81.0 |
| 0127 | 2,014 | 165 | 165.0 |
| 0127 | 2,015 | 92 | 128.5 |
| 0127 | 2,016 | 83 | 113.3 |
| 0127 | 2,017 | 92 | 108.0 |
| 0127 | 2,018 | 69 | 84.0 |
| 0127 | 2,019 | 107 | 87.8 |
| 0127 | 2,020 | 57 | 81.2 |
| 0127 | 2,021 | 39 | 68.0 |
| 0127 | 2,022 | 97 | 75.0 |
| 0127 | 2,023 | 43 | 59.0 |
| 0128 | 2,015 | 18 | 18.0 |
| 0128 | 2,016 | 19 | 18.5 |
| 0128 | 2,017 | 41 | 26.0 |
| 0128 | 2,018 | 20 | 24.5 |
| 0128 | 2,019 | 25 | 26.2 |
| 0128 | 2,020 | 30 | 29.0 |
| 0128 | 2,021 | 22 | 24.2 |
| 0128 | 2,022 | 32 | 27.2 |
| 0128 | 2,023 | 24 | 27.0 |
| 0129 | 2,014 | 40 | 40.0 |
| 0129 | 2,015 | 28 | 34.0 |
| 0129 | 2,016 | 15 | 27.7 |
| 0129 | 2,017 | 22 | 26.2 |
| 0130 | 2,021 | 42 | 42.0 |
| 0130 | 2,022 | 131 | 86.5 |
| 0130 | 2,023 | 116 | 96.3 |
| 0131 | 2,022 | 18 | 18.0 |
| 0131 | 2,023 | 12 | 15.0 |
| 0132 | 2,016 | 37 | 37.0 |
| 0132 | 2,017 | 20 | 28.5 |
| 0132 | 2,018 | 12 | 23.0 |
| 0132 | 2,019 | 32 | 25.2 |
| 0132 | 2,021 | 19 | 20.8 |
| 0134 | 2,014 | 125 | 125.0 |
| 0134 | 2,015 | 157 | 141.0 |
| 0134 | 2,016 | 109 | 130.3 |
| 0134 | 2,017 | 110 | 125.2 |
| 0134 | 2,018 | 163 | 134.8 |
| 0134 | 2,019 | 132 | 128.5 |
| 0134 | 2,020 | 21 | 106.5 |
| 0134 | 2,021 | 30 | 86.5 |
| 0134 | 2,022 | 57 | 60.0 |
| 0134 | 2,023 | 22 | 32.5 |
| 0137 | 2,019 | 273 | 273.0 |
| 0137 | 2,020 | 143 | 208.0 |
| 0137 | 2,021 | 211 | 209.0 |
| 0137 | 2,022 | 175 | 200.5 |
| 0137 | 2,023 | 41 | 142.5 |
| 0140 | 2,014 | 27 | 27.0 |
| 0140 | 2,015 | 32 | 29.5 |
| 0140 | 2,016 | 26 | 28.3 |
| 0140 | 2,017 | 38 | 30.8 |
| 0140 | 2,018 | 38 | 33.5 |
| 0140 | 2,019 | 23 | 31.2 |
| 0141 | 2,016 | 26 | 26.0 |
| 0141 | 2,017 | 35 | 30.5 |
| 0141 | 2,018 | 44 | 35.0 |
| 0141 | 2,019 | 48 | 38.2 |
| 0141 | 2,020 | 17 | 36.0 |
| 0141 | 2,021 | 94 | 50.8 |
| 0141 | 2,022 | 101 | 65.0 |
| 0141 | 2,023 | 65 | 69.2 |
| 0142 | 2,021 | 10 | 10.0 |
| 0142 | 2,022 | 49 | 29.5 |
| 0142 | 2,023 | 26 | 28.3 |
| 0143 | 2,015 | 22 | 22.0 |
| 0143 | 2,016 | 20 | 21.0 |
| 0143 | 2,017 | 24 | 22.0 |
| 0143 | 2,018 | 20 | 21.5 |
| 0143 | 2,019 | 35 | 24.8 |
| 0143 | 2,020 | 26 | 26.2 |
| 0143 | 2,021 | 76 | 39.2 |
| 0143 | 2,022 | 112 | 62.2 |
| 0143 | 2,023 | 65 | 69.8 |
| 0144 | 2,015 | 14 | 14.0 |
| 0144 | 2,017 | 14 | 14.0 |
| 0146 | 2,014 | 49 | 49.0 |
| 0146 | 2,015 | 64 | 56.5 |
| 0146 | 2,016 | 47 | 53.3 |
| 0146 | 2,017 | 90 | 62.5 |
| 0146 | 2,018 | 148 | 87.2 |
| 0146 | 2,019 | 107 | 98.0 |
| 0146 | 2,020 | 66 | 102.8 |
| 0146 | 2,021 | 131 | 113.0 |
| 0146 | 2,022 | 167 | 117.8 |
| 0146 | 2,023 | 97 | 115.2 |
| 0147 | 2,014 | 24 | 24.0 |
| 0148 | 2,017 | 10 | 10.0 |
| 0148 | 2,022 | 62 | 36.0 |
| 0148 | 2,023 | 31 | 34.3 |
| 0149 | 2,014 | 85 | 85.0 |
| 0149 | 2,015 | 61 | 73.0 |
| 0149 | 2,016 | 80 | 75.3 |
| 0149 | 2,017 | 121 | 86.8 |
| 0149 | 2,018 | 146 | 102.0 |
| 0149 | 2,019 | 186 | 133.2 |
| 0149 | 2,020 | 54 | 126.8 |
| 0149 | 2,021 | 216 | 150.5 |
| 0149 | 2,022 | 291 | 186.8 |
| 0149 | 2,023 | 110 | 167.8 |
| 0150 | 2,017 | 32 | 32.0 |
| 0150 | 2,018 | 33 | 32.5 |
| 0150 | 2,019 | 15 | 26.7 |
| 0150 | 2,020 | 27 | 26.8 |
| 0150 | 2,021 | 100 | 43.8 |
| 0150 | 2,022 | 100 | 60.5 |
| 0150 | 2,023 | 43 | 67.5 |
| 0152 | 2,023 | 15 | 15.0 |
| 0153 | 2,014 | 16 | 16.0 |
| 0153 | 2,015 | 18 | 17.0 |
| 0153 | 2,017 | 18 | 17.3 |
| 0153 | 2,018 | 10 | 15.5 |
| 0153 | 2,019 | 15 | 15.2 |
| 0153 | 2,021 | 23 | 16.5 |
| 0153 | 2,022 | 21 | 17.2 |
| 0153 | 2,023 | 22 | 20.2 |
| 0156 | 2,014 | 22 | 22.0 |
| 0156 | 2,015 | 22 | 22.0 |
| 0156 | 2,016 | 46 | 30.0 |
| 0156 | 2,017 | 31 | 30.2 |
| 0156 | 2,019 | 17 | 29.0 |
| 0156 | 2,022 | 10 | 26.0 |
| 0157 | 2,014 | 60 | 60.0 |
| 0157 | 2,015 | 118 | 89.0 |
| 0157 | 2,016 | 98 | 92.0 |
| 0157 | 2,017 | 152 | 107.0 |
| 0157 | 2,018 | 148 | 129.0 |
| 0157 | 2,019 | 133 | 132.8 |
| 0157 | 2,020 | 69 | 125.5 |
| 0157 | 2,021 | 43 | 98.2 |
| 0157 | 2,022 | 103 | 87.0 |
| 0157 | 2,023 | 67 | 70.5 |
| 0159 | 2,014 | 76 | 76.0 |
| 0159 | 2,015 | 86 | 81.0 |
| 0159 | 2,016 | 62 | 74.7 |
| 0159 | 2,017 | 56 | 70.0 |
| 0160 | 2,014 | 33 | 33.0 |
| 0160 | 2,016 | 33 | 33.0 |
| 0161 | 2,017 | 34 | 34.0 |
| 0161 | 2,018 | 30 | 32.0 |
| 0161 | 2,019 | 52 | 38.7 |
| 0161 | 2,020 | 25 | 35.2 |
| 0161 | 2,021 | 21 | 32.0 |
| 0162 | 2,022 | 12 | 12.0 |
| 0163 | 2,017 | 43 | 43.0 |
| 0164 | 2,015 | 18 | 18.0 |
| 0164 | 2,016 | 21 | 19.5 |
| 0164 | 2,017 | 41 | 26.7 |
| 0164 | 2,018 | 29 | 27.2 |
| 0164 | 2,019 | 22 | 28.2 |
| 0164 | 2,020 | 16 | 27.0 |
| 0164 | 2,021 | 46 | 28.2 |
| 0164 | 2,022 | 104 | 47.0 |
| 0164 | 2,023 | 61 | 56.8 |
| 0165 | 2,014 | 38 | 38.0 |
| 0165 | 2,015 | 31 | 34.5 |
| 0165 | 2,016 | 26 | 31.7 |
| 0165 | 2,018 | 20 | 28.8 |
| 0165 | 2,019 | 15 | 23.0 |
| 0165 | 2,021 | 15 | 19.0 |
| 0165 | 2,022 | 46 | 24.0 |
| 0165 | 2,023 | 19 | 23.8 |
| 0167 | 2,014 | 241 | 241.0 |
| 0167 | 2,015 | 256 | 248.5 |
| 0167 | 2,016 | 302 | 266.3 |
| 0167 | 2,017 | 306 | 276.2 |
| 0167 | 2,018 | 239 | 275.8 |
| 0167 | 2,019 | 344 | 297.8 |
| 0167 | 2,020 | 169 | 264.5 |
| 0167 | 2,021 | 186 | 234.5 |
| 0167 | 2,022 | 206 | 226.2 |
| 0167 | 2,023 | 186 | 186.8 |
| 0168 | 2,014 | 14 | 14.0 |
| 0170 | 2,014 | 184 | 184.0 |
| 0170 | 2,015 | 214 | 199.0 |
| 0170 | 2,016 | 305 | 234.3 |
| 0170 | 2,017 | 325 | 257.0 |
| 0170 | 2,018 | 359 | 300.8 |
| 0170 | 2,019 | 378 | 341.8 |
| 0170 | 2,020 | 174 | 309.0 |
| 0170 | 2,021 | 291 | 300.5 |
| 0170 | 2,022 | 311 | 288.5 |
| 0170 | 2,023 | 136 | 228.0 |
| 0171 | 2,014 | 131 | 131.0 |
| 0171 | 2,015 | 174 | 152.5 |
| 0171 | 2,016 | 112 | 139.0 |
| 0171 | 2,017 | 97 | 128.5 |
| 0171 | 2,018 | 135 | 129.5 |
| 0171 | 2,019 | 152 | 124.0 |
| 0171 | 2,020 | 52 | 109.0 |
| 0171 | 2,021 | 117 | 114.0 |
| 0171 | 2,022 | 103 | 106.0 |
| 0171 | 2,023 | 28 | 75.0 |
| 0172 | 2,014 | 29 | 29.0 |
| 0172 | 2,015 | 54 | 41.5 |
| 0172 | 2,016 | 28 | 37.0 |
| 0172 | 2,017 | 50 | 40.2 |
| 0172 | 2,018 | 41 | 43.2 |
| 0172 | 2,019 | 45 | 41.0 |
| 0172 | 2,020 | 12 | 37.0 |
| 0172 | 2,021 | 44 | 35.5 |
| 0172 | 2,022 | 46 | 36.8 |
| 0174 | 2,014 | 363 | 363.0 |
| 0174 | 2,015 | 507 | 435.0 |
| 0174 | 2,016 | 297 | 389.0 |
| 0174 | 2,017 | 420 | 396.8 |
| 0174 | 2,018 | 321 | 386.2 |
| 0174 | 2,019 | 12 | 262.5 |
| 0175 | 2,023 | 26 | 26.0 |
| 0176 | 2,017 | 36 | 36.0 |
| 0176 | 2,018 | 49 | 42.5 |
| 0176 | 2,019 | 36 | 40.3 |
| 0176 | 2,020 | 93 | 53.5 |
| 0176 | 2,021 | 140 | 79.5 |
| 0176 | 2,022 | 49 | 79.5 |
| 0176 | 2,023 | 33 | 78.8 |
| 0177 | 2,014 | 20 | 20.0 |
| 0177 | 2,015 | 22 | 21.0 |
| 0177 | 2,016 | 18 | 20.0 |
| 0177 | 2,017 | 29 | 22.2 |
| 0177 | 2,018 | 29 | 24.5 |
| 0177 | 2,021 | 17 | 23.2 |
| 0177 | 2,022 | 13 | 22.0 |
| 0177 | 2,023 | 55 | 28.5 |
| 0178 | 2,017 | 13 | 13.0 |
| 0178 | 2,018 | 30 | 21.5 |
| 0179 | 2,014 | 14 | 14.0 |
| 0180 | 2,023 | 22 | 22.0 |
| 0181 | 2,023 | 13 | 13.0 |
| 0182 | 2,014 | 122 | 122.0 |
| 0182 | 2,015 | 77 | 99.5 |
| 0182 | 2,016 | 95 | 98.0 |
| 0182 | 2,017 | 87 | 95.2 |
| 0182 | 2,018 | 84 | 85.8 |
| 0182 | 2,019 | 74 | 85.0 |
| 0182 | 2,020 | 33 | 69.5 |
| 0182 | 2,021 | 41 | 58.0 |
| 0182 | 2,022 | 52 | 50.0 |
| 0182 | 2,023 | 21 | 36.8 |
| 0184 | 2,020 | 14 | 14.0 |
| 0184 | 2,021 | 30 | 22.0 |
| 0184 | 2,022 | 19 | 21.0 |
| 0185 | 2,023 | 97 | 97.0 |
| 0188 | 2,014 | 160 | 160.0 |
| 0188 | 2,015 | 249 | 204.5 |
| 0188 | 2,016 | 193 | 200.7 |
| 0188 | 2,017 | 202 | 201.0 |
| 0188 | 2,018 | 177 | 205.2 |
| 0188 | 2,019 | 181 | 188.2 |
| 0188 | 2,020 | 85 | 161.2 |
| 0189 | 2,014 | 247 | 247.0 |
| 0189 | 2,015 | 236 | 241.5 |
| 0189 | 2,016 | 177 | 220.0 |
| 0189 | 2,017 | 56 | 179.0 |
| 0190 | 2,014 | 69 | 69.0 |
| 0190 | 2,015 | 82 | 75.5 |
| 0190 | 2,016 | 104 | 85.0 |
| 0190 | 2,017 | 83 | 84.5 |
| 0190 | 2,018 | 105 | 93.5 |
| 0190 | 2,019 | 135 | 106.8 |
| 0190 | 2,020 | 47 | 92.5 |
| 0190 | 2,021 | 58 | 86.2 |
| 0190 | 2,022 | 65 | 76.2 |
| 0190 | 2,023 | 18 | 47.0 |
| 0191 | 2,014 | 33 | 33.0 |
| 0191 | 2,015 | 36 | 34.5 |
| 0191 | 2,016 | 21 | 30.0 |
| 0191 | 2,017 | 22 | 28.0 |
| 0191 | 2,018 | 29 | 27.0 |
| 0192 | 2,014 | 64 | 64.0 |
| 0192 | 2,015 | 84 | 74.0 |
| 0192 | 2,016 | 106 | 84.7 |
| 0192 | 2,017 | 63 | 79.2 |
| 0192 | 2,018 | 139 | 98.0 |
| 0192 | 2,019 | 96 | 101.0 |
| 0192 | 2,020 | 10 | 77.0 |
| 0192 | 2,021 | 12 | 64.2 |
| 0192 | 2,022 | 49 | 41.8 |
| 0192 | 2,023 | 37 | 27.0 |
| 0193 | 2,017 | 10 | 10.0 |
| 0193 | 2,023 | 42 | 26.0 |
| 0195 | 2,021 | 19 | 19.0 |
| 0195 | 2,022 | 147 | 83.0 |
| 0195 | 2,023 | 71 | 79.0 |
| 0196 | 2,014 | 46 | 46.0 |
| 0196 | 2,015 | 74 | 60.0 |
| 0196 | 2,016 | 87 | 69.0 |
| 0196 | 2,017 | 70 | 69.2 |
| 0196 | 2,018 | 114 | 86.2 |
| 0196 | 2,019 | 103 | 93.5 |
| 0196 | 2,020 | 38 | 81.2 |
| 0196 | 2,021 | 39 | 73.5 |
| 0196 | 2,022 | 14 | 48.5 |
| 0196 | 2,023 | 11 | 25.5 |
| 0197 | 2,014 | 164 | 164.0 |
| 0197 | 2,015 | 163 | 163.5 |
| 0197 | 2,016 | 128 | 151.7 |
| 0197 | 2,017 | 200 | 163.8 |
| 0197 | 2,018 | 192 | 170.8 |
| 0197 | 2,019 | 168 | 172.0 |
| 0197 | 2,020 | 52 | 153.0 |
| 0197 | 2,021 | 60 | 118.0 |
| 0197 | 2,022 | 56 | 84.0 |
| 0197 | 2,023 | 40 | 52.0 |
| 0198 | 2,014 | 59 | 59.0 |
| 0198 | 2,015 | 37 | 48.0 |
| 0198 | 2,016 | 19 | 38.3 |
| 0198 | 2,017 | 40 | 38.8 |
| 0198 | 2,018 | 11 | 26.8 |
| 0198 | 2,019 | 10 | 20.0 |

**Figure S1.**

**Figure S2.** Heatmap of Bonferroni-Adjusted p-values for Pairwise Comparisons Between Hospital Volume Quartiles (I–IV) for Postoperative Complication Rates Following Sleev Gastrectomy
